# Role of Enzyme and Active Site Conformational Dynamics in the Catalysis by α-Amylase Explored with QM/MM Molecular Dynamics

**DOI:** 10.1021/acs.jcim.2c00691

**Published:** 2022-07-26

**Authors:** Rui P. P. Neves, Pedro A. Fernandes, Maria J. Ramos

**Affiliations:** LAQV@REQUIMTE, Departamento de Química e Bioquímica, Faculdade de Ciências, Universidade do Porto, Rua do Campo Alegre s/n, 4169-007 Porto, Portugal

## Abstract

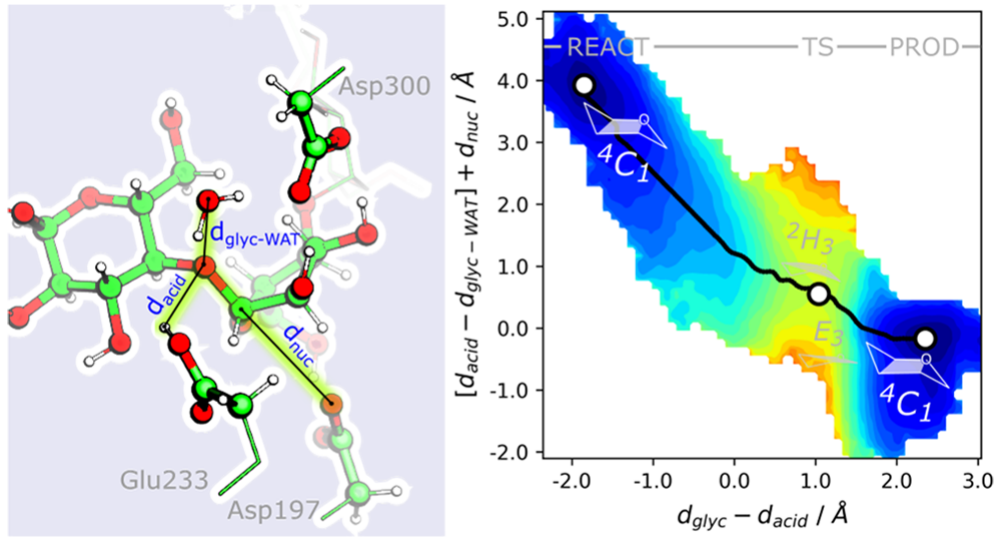

We assessed enzyme:substrate conformational dynamics and the rate-limiting glycosylation step of a human pancreatic α-amylase:maltopentose complex. Microsecond molecular dynamics simulations suggested that the distance of the catalytic Asp197 nucleophile to the anomeric carbon of the buried glucoside is responsible for most of the enzyme active site fluctuations and that both Asp197 and Asp300 interact the most with the buried glucoside unit. The buried glucoside binds either in a ^4^*C*_1_ chair or ^2^*S*_*O*_ skew conformations, both of which can change to TS-like conformations characteristic of retaining glucosidases. Starting from four distinct enzyme:substrate complexes, umbrella sampling quantum mechanics/molecular mechanics simulations (converged within less than 1 kcal·mol^–1^ within a total simulation time of 1.6 ns) indicated that the reaction occurrs with a Gibbs barrier of 13.9 kcal·mol ^–1^, in one asynchronous concerted step encompassing an acid–base reaction with Glu233 followed by a loose S_N_2-like nucleophilic substitution by the Asp197. The transition state is characterized by a ^2^*H*_3_ half-chair conformation of the buried glucoside that quickly changes to the *E*_3_ envelope conformation preceding the attack of the anomeric carbon by the Asp197 nucleophile. Thermodynamic analysis of the reaction supported that a water molecule tightly hydrogen bonded to the glycosidic oxygen of the substrate at the reactant state (∼1.6 Å) forms a short hydrogen bond with Glu233 at the transition state (∼1.7 Å) and lowers the Gibbs barrier in over 5 kcal·mol^–1^. The resulting Asp197-glycosyl was mostly found in the ^4^*C*_1_ conformation, although the more endergonic *B*_3,*O*_ conformation was also observed. Altogether, the combination of short distances for the acid–base reaction with the Glu233 and for the nucleophilic attack by the Asp197 nucleophile and the availability of water within hydrogen bonding distance of the glycosidic oxygen provides a reliable criteria to identify reactive conformations of α-amylase complexes.

## Introduction

Despite the significant progress on the study of enzyme:substrate interactions, the kinetics and thermodynamics of enzyme catalysis or the reaction mechanisms underlying the catalytic power of enzymes,^1−4^ the intricacies of enzyme efficiency and catalysis remain an active field of research and discussion. In particular, the study of reaction mechanisms using computational methods has disclosed relevant molecular details, which potentially lead to structure–thermodynamics relations that can be rationalized under a rigorous physicochemical theoretical framework.^3−7^ In fact, the computational study of the reaction mechanism of enzymes such as lysozyme or human carbonic anhydrase-I^8,9^ led to one of the most accepted hypothesis for the origin of the catalytic power of enzymes among computational biochemists, proposed by Warshel and co-workers, which states that enzymes encompass a preorganized network of dipoles that interacts favorably with transition states and lowers the energy barriers of the catalyzed chemical reactions.^10,11^ According to the proposal, the low rate of chemical reactions in homogeneous media is a result of the high energy that is required to reorient the dipoles of the medium to stabilize the transition state of the chemical reaction.^9,11^

Alongside the important developments in the origin of the catalytic power of enzymes, and together with the increasing availability of computational resources and more efficient exa-scalable codes,^12,13^ it has become clear that the computational simulation of enzyme systems, and their catalyzed reactions in particular, plays now a pivotal role in the rational design of enzymes as efficient biocatalysts for the future generation of sustainable catalysts.^14−18^

Glycosidases are among the most proficient enzyme catalysts in nature, alongside enzymes such as phosphatases or phosphodiesterases.^19,20^ Since proficient enzymes interact strongly with transition states from their catalyzed reactions, their activity can be enhanced upon rationalization of enzyme:substrate interactions along every stage of their catalytic cycle. In particular, glycosidases can be enhanced by a factor of up to 10^17^-fold, and they are important candidates for transition state analogue-based inhibitor design,^21,22^ as well as appealing bioengineering targets.^23,24^

The α-amylase is one of the most studied glycosidases, with important clinical and industrial applications known to date. In mammals, it is responsible for the cleavage of α(1–4) glycosidic bonds in the amylose component of starch, and consequently a primary partner in glucose production for energy acquisition. Consequently, it is also a major target for the development of new inhibitors to treat type-II diabetes.^25,26^ In the microbial world, α-amylases have a broader range of substrate specificities, from amylopectins, cyclodextrins, or glycogen to the starch parent substrate,^27^ which grants them wide applicability in industry, namely in sugar, textile, baking, paper, and brewing industries.^28^

The reaction mechanism of α-amylase has been characterized for pancreatic α-amylase and the starch substrate, where it was shown to occur through a concomitant acid–base reaction and a nucleophilic substitution, involving the catalytic Glu233 and Asp197, to hold a glycosylated enzyme intermediate and release an aglycone; and finally a hydrolysis of the glycosylated enzyme by a water molecule, which is activated by Glu233.^29,30^ Even though the computational kinetic results could reproduce the activation energy, obtained from experimentally determined *k*_*cat*_(31) under the transition state theory framework (14.8 vs 14.4 kcal·mol^–1^),^32^ to our knowledge, there are no studies exploring the reaction space of α-amylase in a proper thermodynamic ensemble.

Early work by Pinto et al., conducted in our group, using hybrid quantum mechanics/molecular mechanics (QM/MM) methods to study the reaction mechanism of human pancreatic α-amylase, identified the glycosylation step as the rate-limiting step of the reaction.^29^ The involved transition state occurred in a dissociative manner, with a concerted late formation of the glycosidic bond with the nucleophilic Asp197 upon cleavage of the substrate’s glycosidic bond, assisted by the Glu233 acid. These observations agreed with other studies in glycosidases.^33,34^ In the same study, the high solvent accessibility at the enzyme’s active site was pointed as a key aspect to achieve full catalysis: not only a water molecule was responsible for an increase in acidity of the Glu233 but also another water molecule was crucial to perform the final hydrolysis step and complete catalysis, restoring the enzyme’s native organization.

More recently, an adiabatic mapping multi-PES QM/MM study by Santos-Martins et al., also conducted in our group, characterized the glycosylation step in 18 conformations of α-amylase and further emphasized the role of the water molecule in the rate-limiting step, showing that a hydrogen bond from a water molecule to the catalytic Glu233 was decisive for favorable catalysis.^35^ The combination of these results and a 100 ns conventional molecular dynamics (cMD), where a volatile hydrogen bond between a water from the bulk and the Glu233 acid was observed, led the authors to infer that catalytically competent enzyme:substrate conformations are in fact short-lived,^35^ further supporting that specific instantaneous active site arrangements are responsible for triggering catalysis by the enzyme.^2,35,36^ Another atomistic study of the glycosidic cleavage step by the psychrophilic α-amylase from *Pseudoalteromonas haloplankti*, led by Kosugi and Hayashi, also pointed out that the reaction was favored by the fast localized reorganization of the active site and by the slow reorganization of a loop adjacent to the binding site at the nanosecond-time scale.^37^ On the other hand, the organization of the active site of α-amylase and the resulting distorted chair conformation of the substrate still reveals several charge-complementarity residues that support the dominating electrostatic preorganization paradigm,^3,10,32^ namely the relative position of the catalytic Glu233 and Asp197 to the scissile glycosidic bond, the concomitant interaction of the nucleophilic Asp197 with hydroxyl groups of the retained substrate in a *syn* orientation to the nucleophilic attack, or the Asp300 anchoring the hydroxyl groups of the retained substrate in an *anti* orientation.^29,38^

### Objective of the Present Work

In this work, we aim to study the enzyme:substrate conformational dynamics through microsecond cMD simulations, and the rate-limiting chemical step through extended QM/MM MD. Hence, we elaborate a comprehensive discussion on the atomistic determinants of the catalysis by α-amylase.

**Figure 1:**
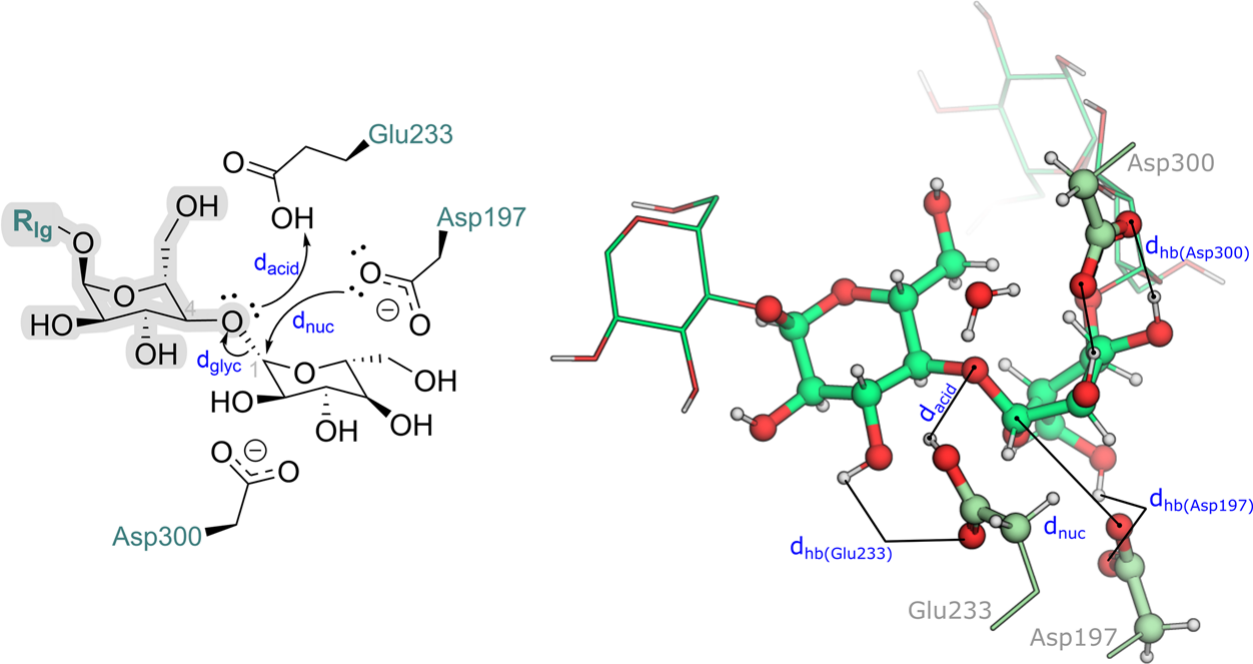
Left: Schematic representation of the glycosylation mechanism of α-amylase with the main distances highlighted in blue. Right: Representation of a minimal active site of α-amylase and the distances analyzed in this study. Atoms represented in ball-and-stick were used in QM calculations. For MM MD simulations, only heavy atoms were considered to calculate interatomic distances.

From the analysis of the most relevant interactions previously identified (Figure 1),^29,35^ we discuss: the implications of the active site organization in the enzyme:substrate complex for the substrate binding and for the thermodynamics of the glycosylation step. Ultimately, we present a focused discussion on the role of active site electrostatics and enzyme conformational dynamics in enzyme catalysis, taking α-amylase as a case study.

## Methods

### Parameterizing the Enzyme:Substrate Model

We picked starting structures based on the criteria of: minimal modeling from the X-ray structure of human pancreatic α-amylase (PDB ID: 1CPU),^39^ and the spread of activation energies determined by the authors of ref (35). A total of 4 models were built based on the coordinates from an X-ray initial structure (PDB ID: 1CPU) and the coordinates at 51.6, 68.7, and 85.7 ns from the 100 ns cMD simulation of ref (35) (here labeled as **A**, **B**, and **C**). The latter correspond to the highest, lowest and in-between activation energies for the glycosylation step of α-amylase as determined from previous work (28.6, 9.3, and 20.1 kcal·mol^–1^, respectively). In such a way, we guarantee that we started from nonequivalent reactive enzyme:substrate complexes that led to very different activation energies, despite their structural similarities at the active site. All protonations were assigned as in ref (35) and all disulfide bonds were inspected. The ff99SB force-field was used for all amino acids of the α-amylase model and calcium and chloride counterions, the GLYCAM_06h force-field was used to describe the maltopentose substrate (5-GA), and the TIP3P model was used for water solvent molecules. The models were enclosed in a periodic rectangular box with 67,992 atoms. Details of minimization and equilibration are provided in the Supporting Information.

### Microsecond Conventional MD

The Gromacs 2018.3 package^40^ was used to run all calculations. Topology and coordinate files were prepared with the *parmed* python module, as available in AmberTools 18.^41^ An initial annealing simulation was run for 100 ps, with constant *N* and *V*, up to a reference temperature of 310 K, followed by a 2 ns equilibration of the density of the solvent in an *NPT* ensemble. An integration time step of 2 fs was used, using the LINCS algorithm^42,43^ to constrain all hydrogen covalent bonds, and a cutoff of 10 Å was used to define explicit particle–particle electrostatic and Lennard-Jones interactions. Electrostatic interactions beyond 10 Å were treated with the particle-mesh Ewald summation method,^44^ using a cubic interpolation grid and a 1.2 Å Fourier-spacing. The pressure of the system was set to 1 bar, coupled with a 2 ps relaxation time isotropic Berendsen barostat,^45^ and a reference temperature of 310 K was set for the solute and solvent, which were coupled independently with a 0.1 ps relaxation time velocity-rescaling thermostat.^46^ After equilibration, an initial 100 ns cMD was run for each of the four starting models, in an *NPT* ensemble at 1 bar and 310 K, with the isotropic Parrinello–Rahman barostat and the velocity-rescaling thermostat.^46,47^ In order to produce microsecond time scale simulations, up to a total of 2 μs, productions of 100 ns were iteratively carried out after a clustering analysis based on the root-mean-square deviation (RMSd) of the heavy atoms of the active site of α-amylase depicted in Figure 1, right).^35^ Each 100 ns trajectory was spliced every 100 ps for a clustering analysis with the Gromos algorithm,^48^ considering an RMSd cutoff of 1.2 Å from the centroid structure. Every centroid structure farthest from the starting structure, and with an occupancy above 10% out of the 100 ns cMD, was selected for a new round of 100 ns cMD. New velocities were generated from a 310 K Maxwell-distribution and a new equilibration was carried out for every cMD iteration. Whenever Gibbs energies were computed, the data (with size *N*) was binned along  bins, and the Gibbs energy was computed according to the expression , where *k*_*i*_ is the occupation of the bin *i*, and *k*_0_ is the occupation of the most occupied bin.

### Umbrella Sampling QM/MM MD

All QM/MM calculations were carried out with the AMBER 18 package, with QM calculations performed externally by the Orca 4.1.2 software, and using the Born–Oppenheimer approximation.^49,50^ The electrostatic embedding approach, including the MM RESP charges in the QM Hamiltonian, was used to address the interaction between the QM and MM layers, and the link atom approach was used to address the boundary between the QM and MM layers. A set of 70 atoms was defined to undergo QM calculations at the PBE/def2-SVP level (Figure 1, right): the methyl–carboxylic terminus of acidic Glu233, nucleophilic Asp197, and transition state stabilizer Asp300; the glucose monomers involved in the scissile glycosidic bond of the substrate; and a water molecule near the acidic oxygen of Glu233 and the oxygen of the scissile glycosidic bond (previously shown to be important for catalysis). The combination of PBE^51^ and small basis sets has been widely used to study reaction mechanisms of glycosidases,^52−55^ as well as to accurately describe the conformational diversity of several carbohydrate units (including the glucopyranose ring composing the 5-GA substrate),^56,57^ and it was also observed to accurately reproduce geometries for the glycosylation reaction with moderately low computational effort.^58^ The use of single split-valence polarization basis sets was also indicated to reduce overpolarization effects in QM/MM MD simulations.^59^ Hence, we assessed the quality of the combination of PBE and the def2-SVP basis set, which compares with the 6-31G** basis set and was previously employed in QM/MM MD studies of biological and systems,^60,61^ to describe the glycosylation step of α-amylase, using the ONIOM methodology within the electrostatic embedding approach, as implemented in Gaussian 16.^62^ Our results indicated that PBE/def2-SVP:AMBER level of theory reproduced the glycosylation step of α-amylase, having showed results comparable to those by Santos-Martins et al. at a higher level of theory (refer to Tables S1–S3 and Figure S1 in Supporting Information for details).

The energy minimization protocol was carried out with 50 steps of steepest descent search followed by a conjugate gradient search up to 200 cycles of minimization, followed by a short 2 ps MD simulation in the *NPT* ensemble (1 bar and 310 K) in a periodic box. The Langevin thermostat with a collision frequency of 1 ps^–1^, and the Monte Carlo barostat with a relaxation time of 2 ps were employed. Nonbonded interactions were treated explicitly within 9 Å of each particle; beyond that, Coulomb interactions were treated with the particle-mesh Ewald scheme and Lennard-Jones interactions were truncated. An integration step of 1 fs was used to integrate motion equations.

After minimization and equilibration QM/MM calculations, a short 2 ps steered MD^63^ was performed for each system along an RC defined as the difference between the distance of the cleaved scissile bond (*d*_glyc_) and the distance of the hydrogen of the acidic Glu233-OH to the oxygen atom of the scissile bond (*d*_acid_),
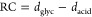


The RC was defined upon inspection of the nuclear vibrations with the highest amplitude from the imaginary frequencies corresponding to the transition states characterized in previous studies.

Umbrella sampling QM/MM MD simulations were performed for the four models along the defined RC. To generate the windows composing each umbrella sampling simulation, a harmonic potential with a force constant of 100–200 kcal·mol^–1^·Å^–2^ was centered every 0.10 Å along the amplitude defined for the RC. Starting conformations for each window were chosen as the ones from the trajectory of the steered MD whose RC value was the closest to the center of the harmonic potential applied in each window, and ran for 10 ps (resulting in a total of 450–500 ps per QM/MM model). A detailed description is provided in Supporting Information (section *Umbrella sampling QM/MM MD* and Figure S2). The data for the four models was then concatenated (total of ∼1.6 ns) and processed with the weighted-histogram analysis method as implemented in the WHAM software, by Grossman et al.^64^ During the QM/MM MD simulations, time-dependent block analysis was carried out in the direct and reverse time series to evaluate the statistical convergence of the resulting potential of mean force (PMF) plots for the glycosidic cleavage step, until a difference below 1 kcal·mol^–1^ was observed between two successive PMFs. Finally, the first 2 ps of each window were considered an equilibration period and discarded (refer to Figure S3 in the Supporting Information for details).

For subsequent analysis, the individual probability of each simulated conformation, *p*_*i*_, was then determined from the WHAM equation,
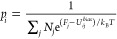
where the denominator is calculated as the sum of the Boltzmann factor of the difference between the reference energy *F*_*j*_ of window *j*, as retrieved from a prior WHAM analysis, and the bias potential *U*_*ij*_^*bias*^ of conformation *i* in window *j*, for every of the *N*_*j*_ conformations in each window. As for the analysis of the MM simulations, projections of the Gibbs energy along different coordinate data sets were calculated after binning the data (with size *N*) along  bins, according to the expression Δ*G* = −*k*_*B*_*T* ln *p*, where *p* corresponds to the accumulated probability of every conformation occupying each bin.

## Results and Discussion

### Enzyme:Substrate Dynamics

We have conducted microsecond MM simulations to assess the organization of the active site of α-amylase, its domain dynamics, and the main interactions involved in substrate binding.

**Figure 2:**
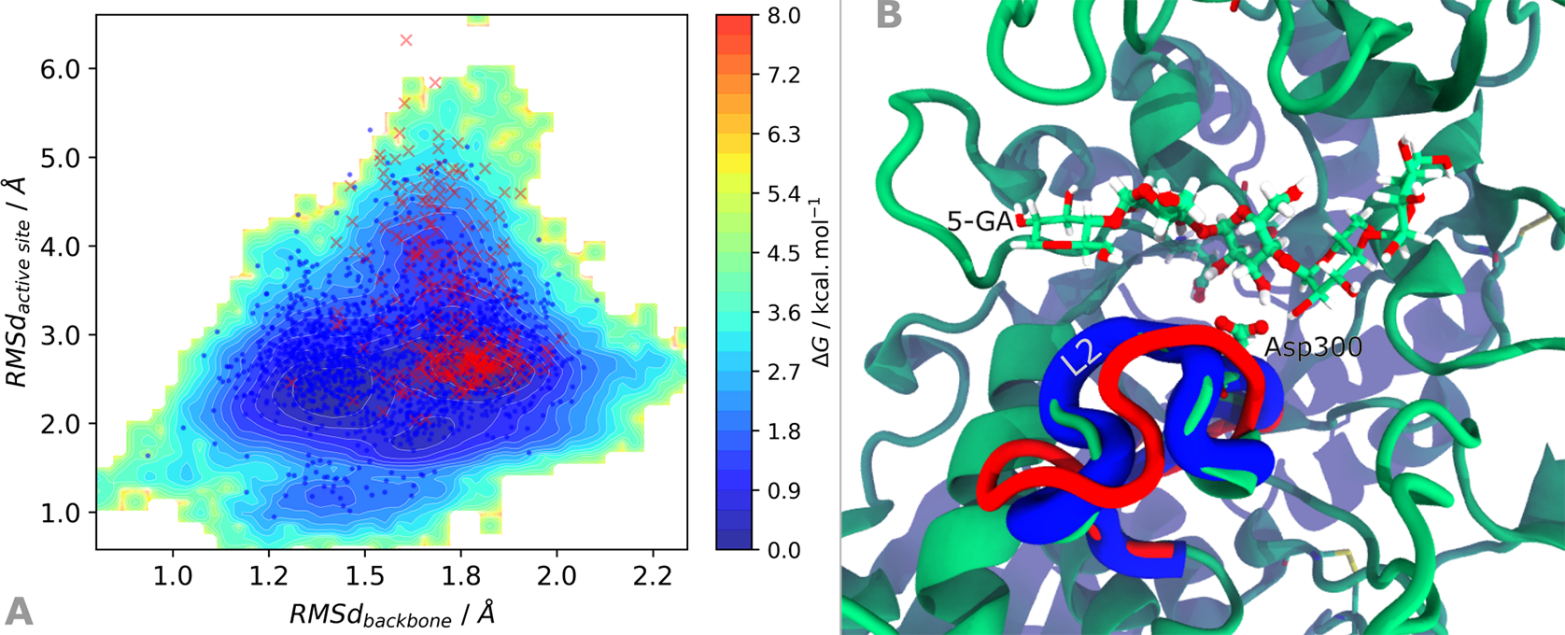
(A) Gibbs energy plot of the 2 μs cMD projected on the space of the RMSd of the backbone and of the active site heavy atoms, with blue dots and red crosses representing the conformations composing the clusters with 85% and 14% occupation after clustering analysis with the backbone atoms of the residues in loop L2. (B) The two distinct representative conformations of the loop L2 are also represented; the 5-GA substrate and the conserved Asp300, relevant for the catalysis, are also highlighted.

The α-amylase exhibits low flexibility when complexed with a maltopentose (5-GA) substrate, on the microsecond time scale, as can be verified by the small variations of the enzyme backbone (below 2.0 Å, Figure 2A) relative to the starting X-ray conformation and the root-mean-square fluctuations for the heavy atoms of each residue (refer to Figure S4 in the Supporting Information). When clustering the simulation by the RMSd of the enzyme’s backbone using a 1.6 Å cutoff, two most representative conformations were identified with occupancies around 85% and 14%. While the most populated cluster closely resembles the starting X-ray conformation, the latter conformation exhibits an alternative conformation of the L2 loop (here composed by residues Phe295 to Thr314, Figure 2B), which was previously identified to stabilize the glycosylation step in the psychrophilic α-amylase, by compressing the transition state.^37^ After clustering the same trajectory using the backbone atoms of the L2 loop using a 2.4 Å cutoff, we again observe that the two most representative clusters show similar occupation (83% and 14%), which suggests that, within the microsecond time scale, the L2 loop is the most flexible structural element of the enzyme:substrate complex.

**Figure 3:**
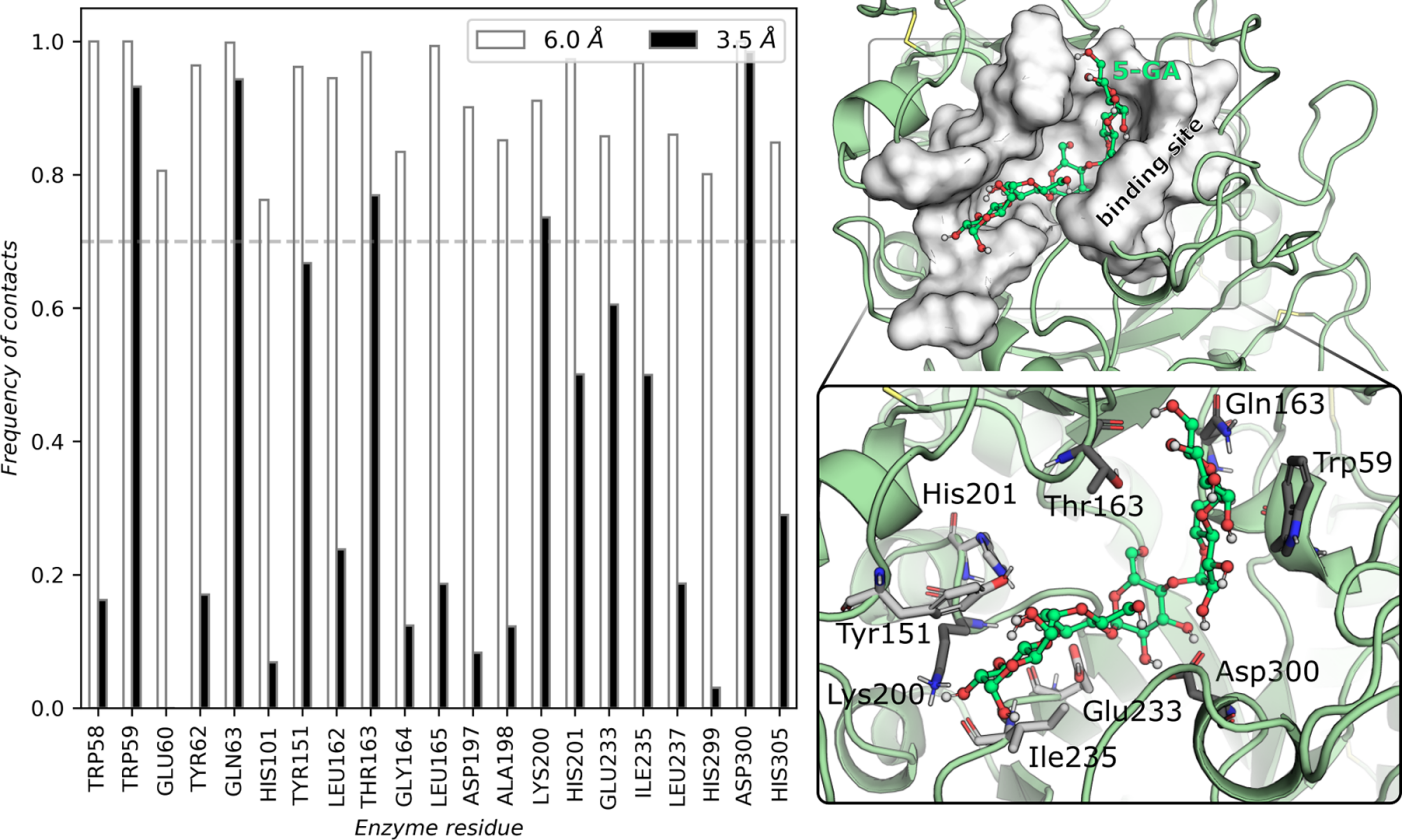
Left, enzyme residues with the most contacts with the 5-GA substrate (>70%) during the 2 μs MD simulations: residues within 3.5 Å interact closely with the substrate and contribute the most for substrate binding, and residues within 6 Å compose the binding pocket of α-amylase. Right, depiction of the binding site of the 5-GA substrate in white surface representation and of the close contacts with the 5-GA substrate (carbons colored in dark gray and light gray, respectively, for residues establishing contacts for longer than 70% and 50% of the MD simulations).

The analysis of the RMSd of the catalytic residues at the enzyme’s active site suggests that the binding of 5-GA to the active site of the enzyme may not be strongly promoted by every catalytic residue in the active site of the enzyme (the RMSd of the catalytic residues and glucoside units subject to cleavage in the active site varies significantly more than that of the backbone, Figure 2A). In fact, binding of the maltopentose substrate (5-GA) to the enzyme occurs mostly through its buried glucoside units, which closely interact with the catalytic Asp300 and residues Trp59 and Gln63, for over 90% of the simulation, whereas the close interactions with the leaving glucoside units are mostly maintained through less than 70% of the simulations (residues Lys200, Tyr151, Glu233, His201, and Leu235 in Figure 3).

In line with previous observations on retaining α-glycoside hydrolases,^53^ such as α-amylase, the Cremer–Pople angle representation of the glucoside that will be subject to glycosylation (Figure S5) indicates that the glucoside is kept in the chair conformation ^4^*C*_1_ for most of the simulation, which is the preactivated conformation for catalysis, but it can also change to the skew conformation ^2^*S*_*O*_ (Δ*G* around +2.7 kcal·mol^–1^). The latter conformation was suggested to favor TS-like conformations with a planar glucoside ring and a strong oxocarbenium-like character.^65,66^

**Figure 4:**
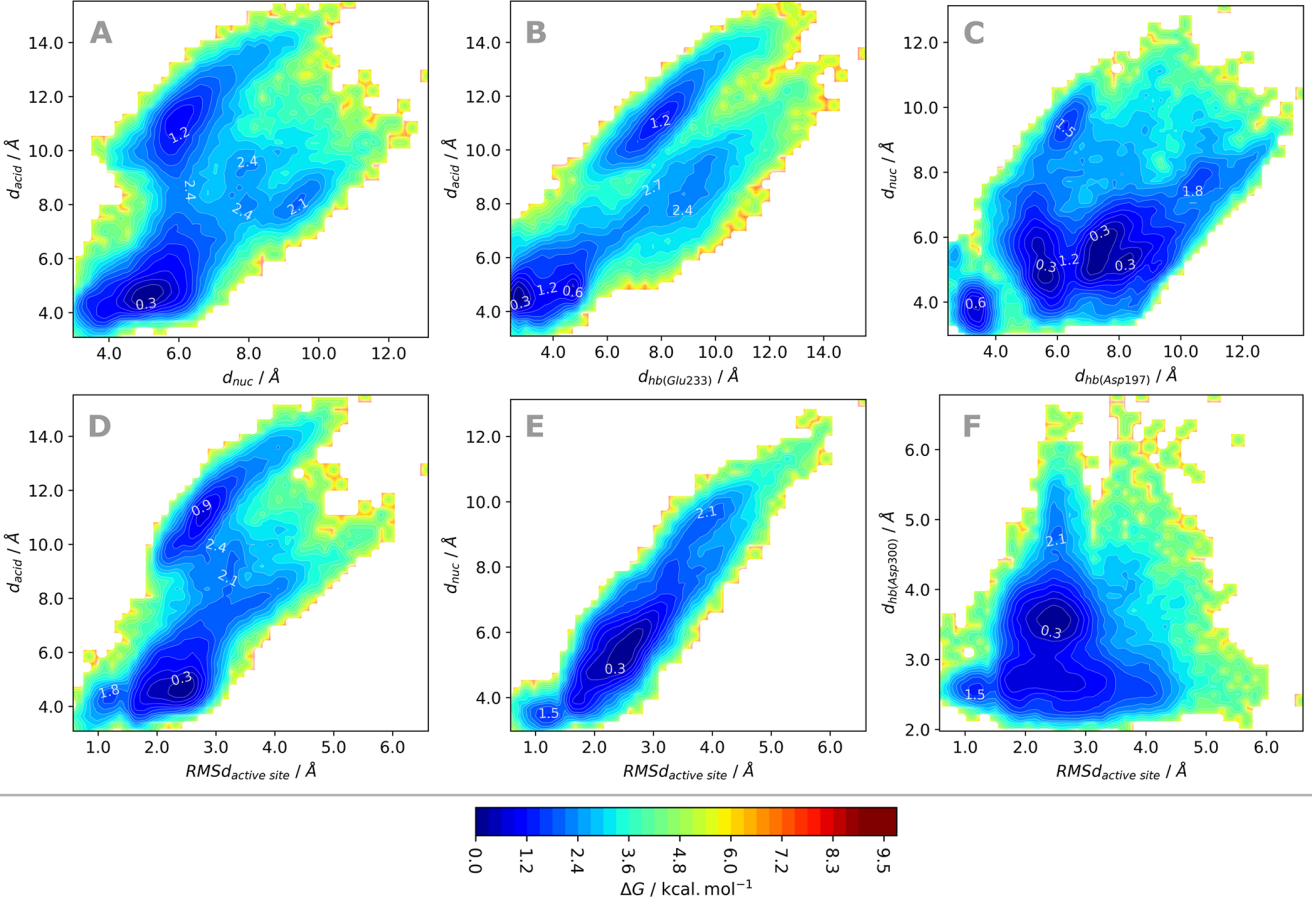
Gibbs energy plot of the 2 μs cMD projected (A) on the space of the two distances describing the formation of new covalent bonds (*d*_acid_ vs *d*_nuc_); (B, C) on the space of the *d*_acid_ and *d*_nuc_ reactive distances against the corresponding anchoring hydrogen bonds established with the glucoside units of the 5-GA substrate, *d*_hb(Glu233)_ and *d*_hb(Asp197)_; and (D–F) on the space of the RMSd of the catalytic residues and glucoside units subject to cleavage plotted against the three most significant distances previously identified as key for the reaction mechanism of α-amylase (*d*_acid_, *d*_nuc_, and *d*_hb(Asp300)_). White-labeled values correspond to the relative Gibbs energies of the regions adjacent to the minima identified as most relevant in each representation.

To better understand the interactions between the 5-GA substrate and the catalytic residues of α-amylase, we analyzed the distances describing the deprotonation of Glu233, the nucleophilic attack of Asp197 and the hydrogen bonding interactions with Asp300 (*d*_acid_, *d*_nuc_, and *d*_hb(Asp300)_ in Figure 1).^29^ The analysis of the relative orientation of Glu233 and Asp197 toward the 5-GA substrate is depicted in Figure 4A. Both these distances span beyond 10 Å in an uncorrelated fashion, describing two clear minima. Nevertheless, the most occupied minimum covers above 20% of the conformational space and it is located on a region where distance metrics suggest that the nucleophilic attack of Asp197 can occur concertedly with the deprotonation of Glu233 (*d*_nuc_ within 4.7–5.5 Å and *d*_acid_ within 4.3–5.0 Å), supporting the concerted reaction hypothesis.^29,37^ Upon comparison of these distances along the two clusters, short distances are available in both, although shorter *d*_nuc_ are more frequent in the cluster that most resembles the X-ray conformation (Figure S6).

Our analysis suggests that the nucleophilic distance from Asp197 to the glycosidic oxygen atom of the substrate (*d*_nuc_) is the most correlated with the RMSd of the active site, which is apparent from the *d*_nuc_ vs RMSD_active site_ plot in Figure 4E and the resemblance of Figure 4, parts A and D, whereas the tight interaction of the Asp300-carboxylate group with the 2,3-OH groups of the buried glucoside unit (*d*_hb(Asp300)_) is conserved regardless of the RMSd of the active site (Figure 4F), contributing actively to the binding of the 5-GA.

The distance of the Glu233-OH to the glycosidic oxygen atom of the substrate (*d*_acid_) varies along the cMD trajectory, as expected by the low accessibility to the glycosidic oxygen provided by the bulk of its bridging glycosidic rings and by its low polarity, which makes it a poor hydrogen bond acceptor. Nevertheless, shorter distances are mostly observed when Glu233 accepts a hydrogen bond from the 3-OH group of the leaving glucoside unit (Figure 4B). The observation supports that the establishment of this hydrogen bond can increase the probability of Glu233 deprotonation by the glycosidic oxygen. The same does not seem to occur for the Asp197 nucleophile, where *d*_nuc_ varies independently from the hydrogen bond between the Asp197 and the 6-OH of the buried glucoside unit (Figure 4C), suggesting that Glu233 may play an important role in proper binding of the leaving glucoside unit, while Asp300 is the most responsible for the binding of the buried glucoside unit.

### The Rate-Limiting Glycosylation Step of α-Amylase

**Figure 5:**
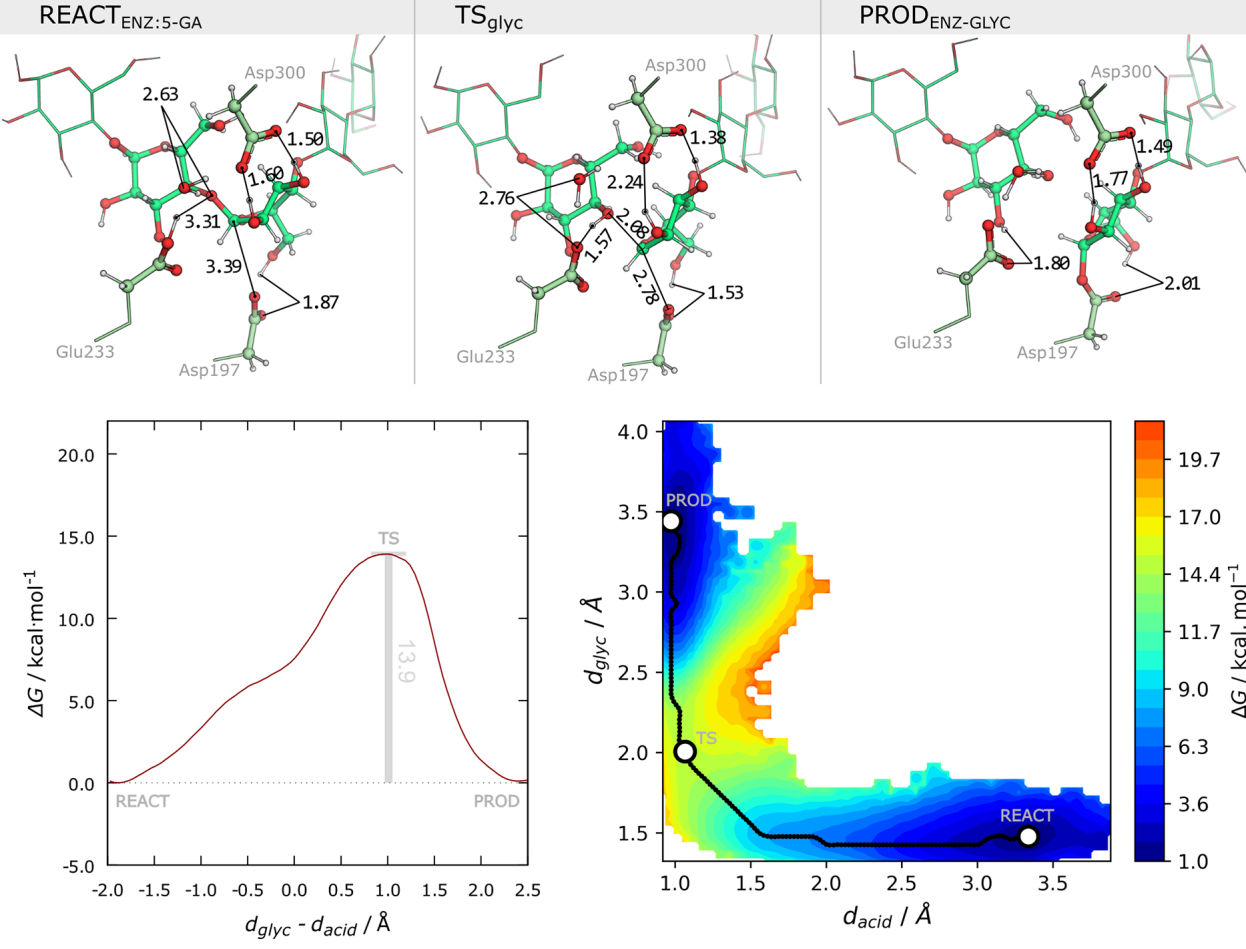
Top panel: representative stationary points for the reaction mechanism of α-amylase glycosylation, from a *k*-means clustering of the 1.6 ns QM/MM MD simulations along the explored reaction coordinate (stretching of the glycosidic bond, *d*_glyc_, and deprotonation of Glu233, *d*_acid_). Highlighted distances (Å) correspond to the most conserved distances at each stationary point of the reaction. Bottom panel: (left) Gibbs energy profile for the accumulated umbrella sampling QM/MM MD simulations of the last 8 ps (2–10 ps) of each starting structure; (right) projection of the Gibbs energy along the coordinates composing the tentative RC, *d*_acid_ and *d*_glyc_, during the equilibrated umbrella sampling QM/MM MD simulations.

Figure 5 (on top) depicts representative stationary points of the glycosylation step retrieved from the accumulated 1.6 ns equilibrated QM/MM MD simulations, upon a clustering analysis of the total simulation with the *k*-means algorithm.

A thermodynamic analysis of the process, as depicted in Figure 5, shows that the glycosylation step of Asp197 in α-amylase occurs in one step with a Gibbs barrier around 13.9 kcal·mol^–1^, and it involves a negligible change in the Gibbs energy of the system upon the formation of the glycosylated Asp197. Although the Gibbs barrier is in very good agreement with experimental and computational determinations, the Gibbs reaction energy differs from that determined in other computational studies, where it was determined to be exergonic.^29,37^

Upon projection of the Gibbs energy along the *d*_acid_ and *d*_glyc_ distances composing the tentative reaction coordinate of the glycosylation step (RC), as depicted in Figure 5 (bottom right), we confirm that, in line with previous work,^29,35^ the acid–base reaction by Glu233 and the release of an aglycone upon the nucleophilic attack of Asp197 occur in a concerted asynchronous manner, with the acid–base reaction preceding the nucleophilic attack by Asp197. A closer analysis at the distances along the representative stationary points in Figure 5 indicates that the most significant changes occur from the reactant to the transition state, with an emphasis on the hydrogen bonds formed between the buried water and both the glycosidic oxygen and the Glu233-OH, as well as those formed with the buried glucoside (between the Asp197-carboxylate and the 6-OH, and the Asp300-carboxylate and 2,3-OH).

The transition state region is characterized by a broad *d*_glyc_ range (from 1.8 to 2.3 Å), and a narrow *d*_acid_ range corresponding to a protonated aglycone, which emphasizes both the loose character of the transition state of the S_N_2 substitution and the asynchronous nature of the glycosylation step. A closer analysis of *d*_nuc_ and the breaking glycosidic bond, *d*_glyc_, for each independent starting conformation, suggests that more dissociative transition states result in higher Gibbs barriers for the process (Figure S7, X-ray and conformation A).

**Figure 6:**
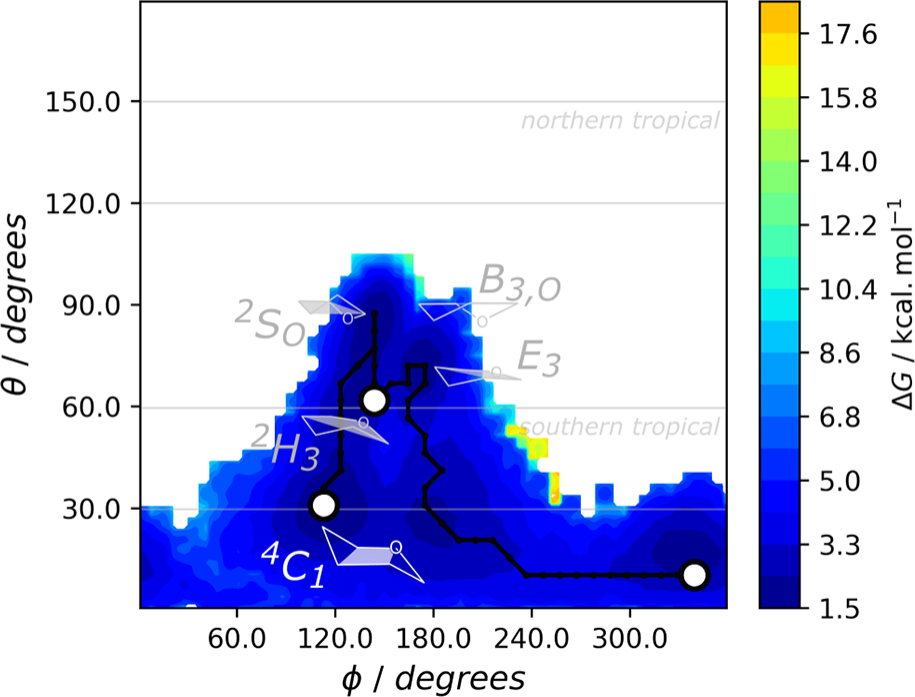
Projection of the Gibbs energy along the Cremer-Pople puckering angles φ and θ for the glucoside undergoing nucleophilic attack by Asp197, during the equilibrated umbrella sampling QM/MM MD simulations. The puckering conformations found for the glucoside are highlighted: chair (^4^*C*_1_), skew (^4^*S*_*O*_), half-chair (^2^*H*_3_), envelope (*E*_3_), and boat (*B*_3,*O*_) conformations.

An analysis of the Cremer–Pople polar coordinates (φ and θ) also shows the effects of ring puckering in the α-d-glucopyranose that is glycosylated by the Asp197. While retaining α-glucosidases, which include the α-amylase here studied, were proposed to follow a ^4^*C*_1_ → [^4^*H*_3_]^‡^→ ^1^*S*_3_ pathway during the glycosylation step,^67^ a recent study by Alonso-Gil et al. observed that in the amylosucrase of *Neisseria polysaccharea*, which also belongs to the same family of α-amylase, the α-d-glucopyranose of the sucrose substrate was kept in the ^4^*C*_1_ chair conformation both at the reactant and glycosylated stages.^53^ These results suggest that not all enzymes in the same family may follow similar ring puckering pathways in the glycosylation step. Consequently, we performed a similar analysis for the glycosylation step of α-amylase, which we summarize in Figure 6.

In line with the results of Alonso-Gil, the ^4^*C*_1_ conformation is also prevalent at the reactant state and for the glycosylated intermediate. In addition, the reactant state may interchange between the ^4^*C*_1_ and ^2^*S*_*O*_ conformations, in line with the results of our cMD simulations. The TS of the glycosylation step then comprises a change in the glucoside ring to the half-chair ^2^*H*_3_ conformation, which rapidly converts into the envelope *E*_3_ conformation where the anomeric carbon is most electrophilic, before holding the prevalent ^4^*C*_1_ conformation once the nucleophilic attack by the Asp197 is concluded. As previously observed by Biarnés et al. in the *Bacillus* 1,3–1,4-β-glucanase,^52^ we observe that the TS does not correspond to the conformation where the anomeric is the most electrophilic. Interestingly, after performing the same analysis for each of the four starting conformations simulated (Figure S8), we observe that those in which the ^2^*S*_*O*_ conformation is dominant led to more endergonic Gibbs reaction energies for the glycosylation step, which were characterized by products both in the *B*_*3,O*_ boat conformation and the prevalent ^4^*C*_1_ chain conformation and to TSs farther from the half-chair/envelope TS-like conformations. Previous electronic structure calculations carried out in isolated pyranose rings by Mayes et al. indicated that the interchange between the ^4^*C*_1_ and ^2^*S*_*O*_ conformations is possible, although the latter should be short-lived and fall either to the ^4^*C*_1_ or *B*_3,*O*_ conformations.^68^ Hence, our higher Gibbs reaction energy is likely the result of the relative abundance of the different conformations of the Asp197-glycosylated product, which could not be reproduced with the conducted adiabatic mapping calculations unless multiple independent QM/MM calculations are performed.^35,69^

#### Hydrogen Bonds during the Glycosylation Step

**Figure 7:**
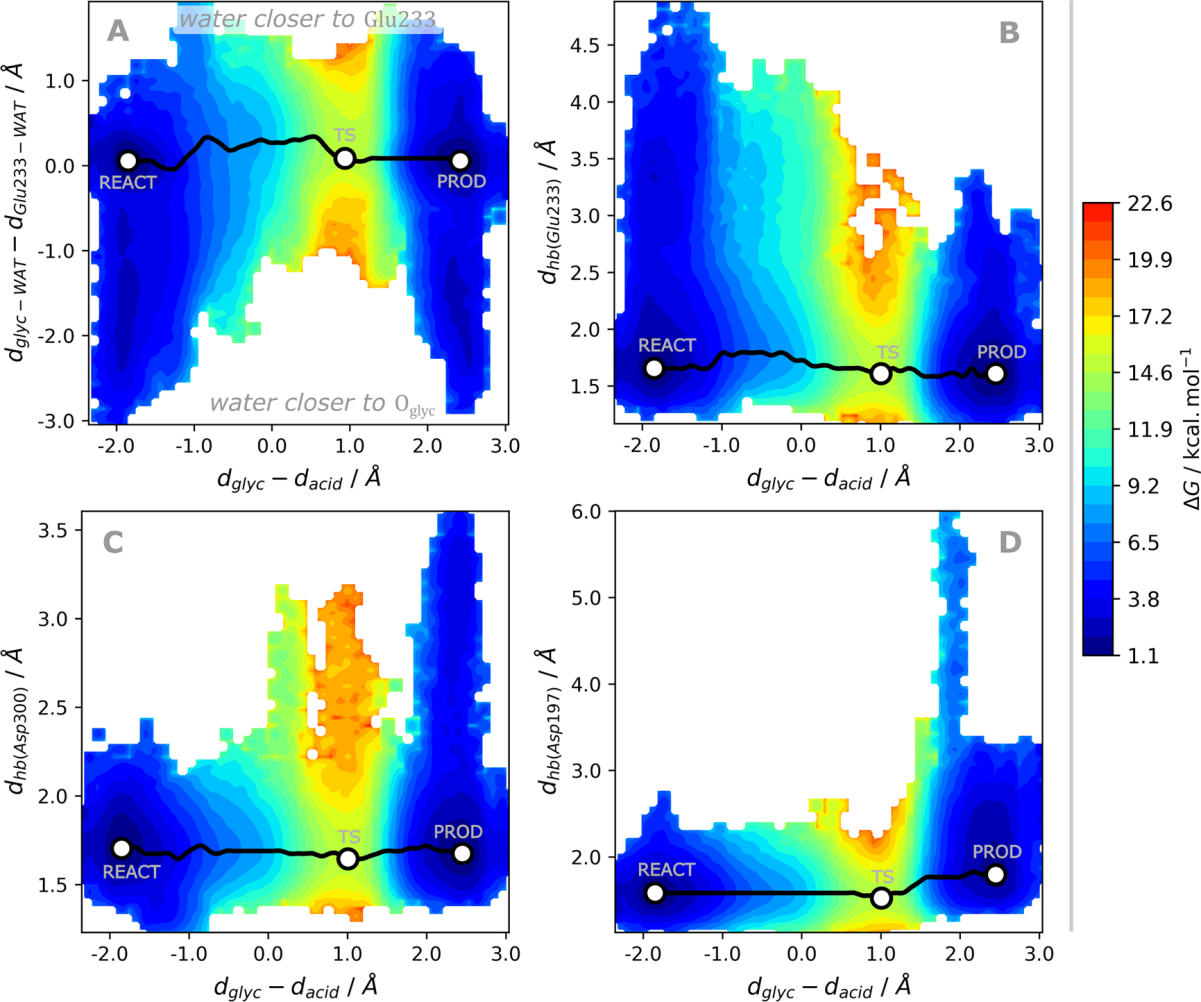
Projection of the Gibbs energy along the coordinates composing the tentative RC, *d*_acid_ and *d*_glyc_, during the equilibrated umbrella sampling QM/MM MD simulations and the main hydrogen bonds taking place.

Regarding Glu233 and Asp197, key for the glycosylation step, a structural analysis suggests that the reaction is favored by the conserved hydrogen bond formed between the Asp197 and the 6-OH of the buried glycosidic unit (Figure 7D), while the hydrogen bond between 3-OH of the leaving glucoside and the Glu233 acid is less prevalent up to the transition state, when the building negative charge in Glu233 strongly favors the hydrogen bond with the leaving glucoside unit (Figure 7B).

Together with the Asp197, the Asp300 establishes the more frequent short hydrogen bonds with the buried glucoside (Figure 7C) and keeps the buried glucoside in place for catalysis. These hydrogen bonds are also significant for transition state stabilization, as longer hydrogen bonds correspond to higher Gibbs energies nearby the transition state region (refer also to Figure S9 for an analysis by starting conformation). The Asp300 also lowers the Gibbs reaction energy, as supported by the estimated energy difference of ∼3 kcal·mol^–1^ required to weaken hydrogen bonds with Asp300 at the product state (see PROD in Figure 7C), and the higher reaction energies observed for the conformations A and C (refer also to Figure S3 in Supporting Information). The fact that this interaction is less prevalent in the product state than in the reactant state may also explain why the glycosylation step involves a negligible Gibbs energy change, even though the release of the resulting aglycone should then be entropically favored, resulting in an overall exergonic process.

The analysis of the proximity of the buried water to the glycosidic oxygen and the Glu233 acid, in Figure 7A (refer also to Figures S8 and S9) indicates that the availability of water solvent nearby the glycosidic oxygen is favored in the reactant state, regardless of the starting conformation. On the other hand, the hydrogen bond to the Glu233 acid is weak in the reactant state, as it varies from 2.5 to 5.0 Å at a cost within less than 2 kcal·mol^–1^, and the interaction becomes more favorable as the nucleophilic attack by Asp197 is performed, which indicates that the water molecule may stabilize the building negative charge of Glu233 upon the formation of the aglycone.

#### Molecular Determinants for the Catalytic Rate

In order to outline the molecular determinants promoting a high or low catalytic rate of α-amylase, we compared the same molecular descriptors for the least favorable reaction (conformation A), whose Gibbs barriers and reaction energies varied from ∼25 to 16 kcal·mol^–1^, against the final equilibrated QM/MM MD simulations.

**Figure 8:**
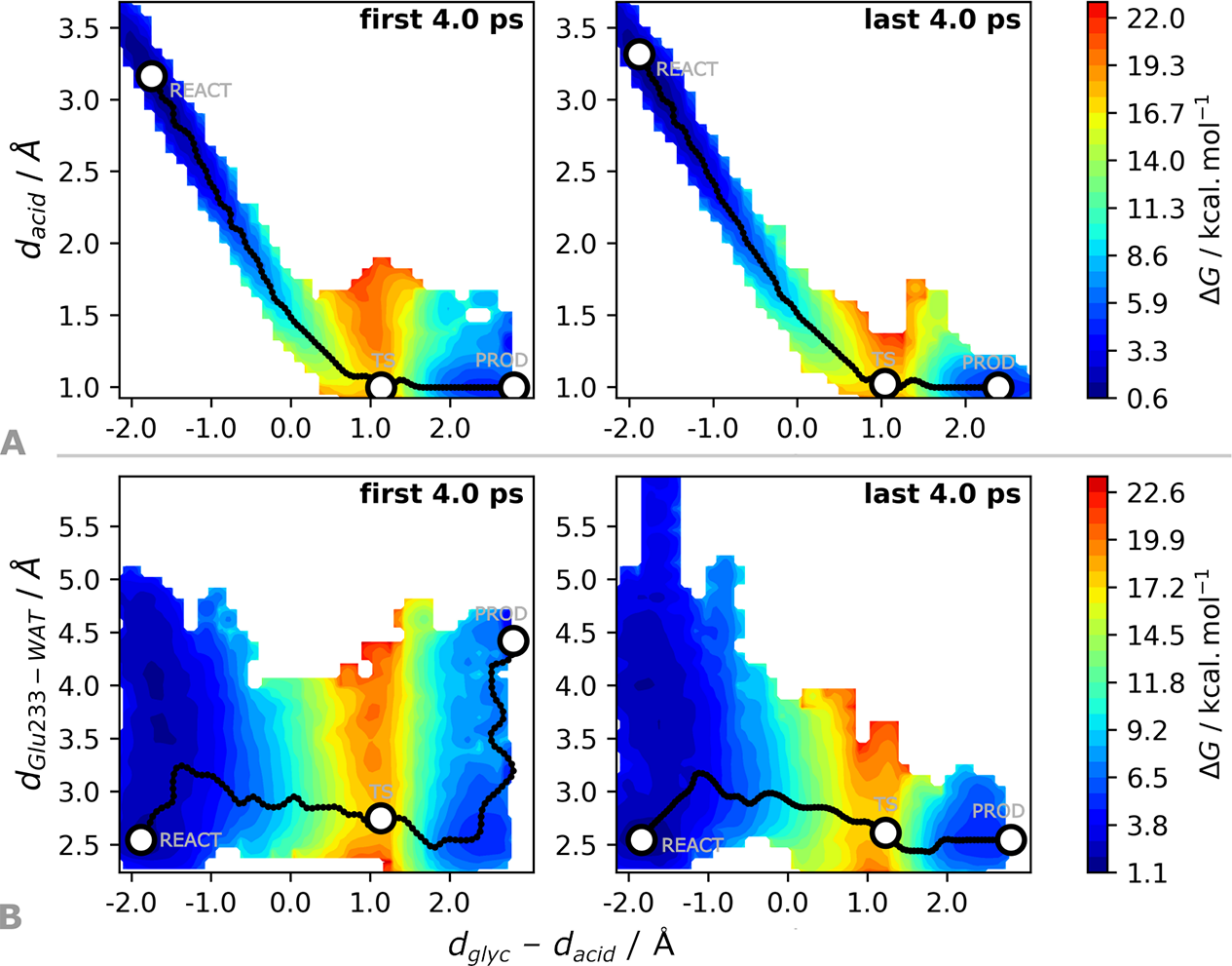
Projection of the Gibbs energy along the coordinates composing the tentative RC, *d*_acid_ and *d*_glyc_, during the umbrella sampling QM/MM MD run for the starting conformation at 51.6 ns (highest energy barrier from ref (35)) and the protonation of the glycosidic oxygen by Glu233 (*d*_acid_, on top) and the distance from the buried water at the active site to the Glu233 acid (*d*_Glu233–WAT_, on bottom).

The most significant difference occurs for the acid–base reaction involving the Glu233, which is most favored when *d*_glyc_ stretches farther than 2 Å. At this stage, the deprotonation of the Glu233 is favored in over 5 kcal·mol^–1^, as shown in Figure 8. Comparing the accumulated first and last 4 ps of the QM/MM MD simulations starting at 51.6 ns, the deprotonation of the Glu233 at the transition state is less frequent for the accumulated data of the initial 4 ps (Figure 8, top). These lower rates of Glu233 deprotonation occur for distances between the nearby buried water and the Glu233 acid (*d*_Glu233–WAT_) above 3.5 Å (Figure 8, bottom), which is also indicated by Figure S10 to result in an increase of the Gibbs barrier by over 5 kcal·mol^–1^.

Previous results from multi-PES QM/MM ONIOM calculations suggested that a water nearby the acidic Glu233 modulates the catalytic rate of α-amylase.^35^ The water would decrease the p*K*_a_ of Glu233 through hydrogen-bonding to the carboxylic acid group and thus promote its deprotonation by the glycosidic oxygen. The authors indicated that lower activation energies should be expected for lower sums of *d*_acid_ + *d*_nuc_ + *d*_Glu233_ at the reactant state, so-called *d*_actsite_, which means that more productive conformations for the glycosylation step would require for: the Glu233 acid to be close to the glycosidic oxygen (short *d*_acid_), the Asp197 nucleophile to be close to the anomeric carbon of the buried glucoside unit (short *d*_nuc_), and a water molecule to be within hydrogen bonding distance of the Glu233 acid (short *d*_Glu233–WAT_).^35^

**Figure 9:**
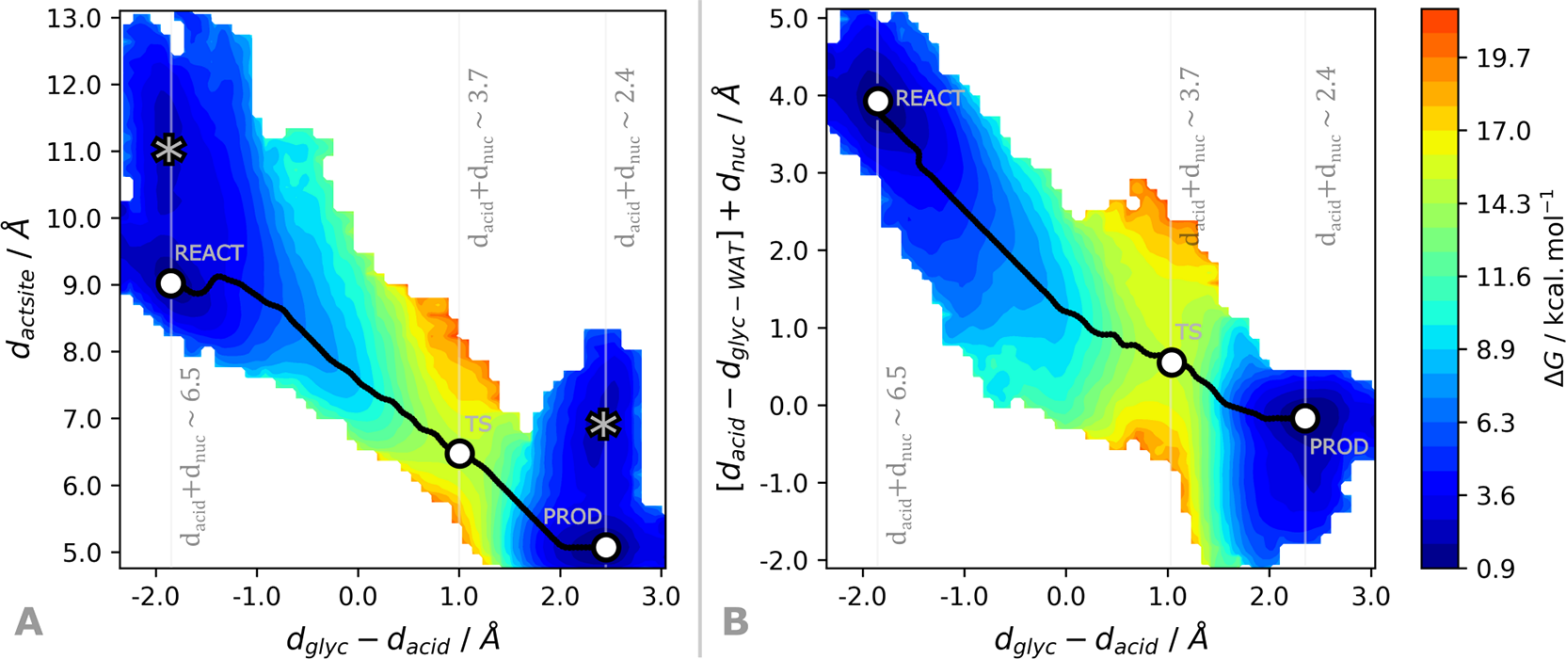
Projection of the Gibbs energy along the collective variable defined by Santos-Martins et al., *d*_actsite_ = *d*_acid_ + *d*_nuc_ + *d*_Glu233–WAT_ (on the left), and *d*_acid_ – *d*_glyc–WAT_ + *d*_nuc_ (on the right). Alternative local minima are shown with gray markers. The sum *d*_acid_ + *d*_nuc_ that is common to both plots is also shown for each of the stationary states of the glycosylation step.

The same representation (left-hand side of Figure 9) indicates that two minima are available for the reactant state (at *d*_actsite_ ≈ 9 and 11 Å), within a barrier below 3 kcal·mol^–1^ and with a Gibbs energy difference of ca. 1 kcal·mol^–1^. Since both *d*_acid_ and *d*_nuc_ vary monotonically during the glycosylation step, this result indicates that a direct hydrogen bond between the buried water and Glu233 is only slightly thermodynamically favored, and it might not be a requisite for productive conformations. Instead, the availability of a water molecule within the first and second solvation spheres of Glu233 (between 3 and 6 Å) is required to promote the formation of a short hydrogen bond between the water and Glu233 at the transition state (*d*_Glu233–WAT_ below 2.6 Å) and consequently lower the Gibbs energy barrier for the reaction.

Our results suggest that the availability of water within hydrogen bonding distance of the glycosidic oxygen, represented by *d*_glyc–WAT_, is a better criterion to characterize productive conformations, together with *d*_acid_ and *d*_nuc_. In fact, the short interaction between the glycosidic oxygen and the water is thermodynamically more favorable than that between the water and the Glu233, as was confirmed in Figure 7A, and does not impair the hydrogen bond formed with the Glu233 toward the transition state. Hence, during the glycosylation step, the hydrogen bond formed between the water and the glycosidic oxygen (*d*_glyc–WAT_) increases as the Glu233 acid becomes closer to the glycosidic oxygen (*d*_acid_) to carry out the acid–base reaction leading to the formation of the transient oxocarbenium glucoside, and the Asp197 nucleophile attacks the anomeric carbon of the resulting oxocarbenium glucoside (*d*_nuc_) leading to the glycosylated Asp197. By combining these three distances, [*d*_acid_ – *d*_glyc-WAT_] + *d*_nuc_, we obtain a monotonic variable with the three clear stationary points characteristic of the glycosylation step (refer to the right-hand side of Figure 9). As a result, we believe that this combination might be a better alternative to quantify productive conformations to carry out the rate-limiting glycosylation step of α-amylase.

We also inspected the behavior of the L2 loop throughout the glycosylation step to assess how it could contribute for catalysis. However, our clustering analysis over the RMSd backbone atoms of the residues comprising the loop indicated that the representative structures of each cluster are similar (0.6 Å) when compared to those from the MM MD simulations (2.4 Å). Since each of our independent QM/MM MD simulations are well below the nanosecond time scale, we cannot infer the contribution of the conformation of the L2 loop for the glycosylation step, although we observe that the glycosylation step could still be carried out efficiently when no appreciable changes were observed in its overall conformation.

Ultimately, we observe that the catalytic rate of α-amylase is ensured by the tight binding of the buried glucoside unit by strong hydrogen bonds from its 2,3,6-OH groups to Asp300 and the nucleophilic Asp197 itself. Then, because of the extended conformation in which α-amylase binds the 5-GA substrate, the glycosidic oxygen bridging the buried and leaving glucosides exhibits a strong ability to accept hydrogen bonds from waters in the bulk solvent, which will lower the p*K*_a_ of the Glu233 acid upon proximity and favor its deprotonation to stabilize the oxyanion resulting from the nucleophilic attack of Asp197 at the anomeric carbon.

## Conclusions

We elaborate on a comprehensive discussion on the atomistic determinants of enzyme catalysis by human pancreatic α-amylase, with quantitative and qualitative detail, by combining extensive conventional and QM/MM MD simulations for a set of distinct enzyme:substrate conformations.

Microsecond MD simulations showed that the α-amylase exhibits a very rigid backbone dynamics, as opposed to that of its active site. In particular, the enzyme was observed to interact more closely with the buried part of the substrate, where the nucleophilic attack by Asp197 occurs, through contacts below 3.5 Å with the catalytic Asp197 and Asp300 and the Trp59, Gln63, Thr163, and Lys200 residues. Despite the tight binding or the buried substrate for over 70% of the simulated time, favorable catalytic distances for the Glu233 acid and the Asp197 (below 5 Å) were only observed for about 20% of the simulated time. The catalytic distance describing the nucleophilic attack of Asp197 to the anomeric carbon of the buried glucoside was observed to be the most affected by changes in the active site, which suggests that the glycosylation step can be regulated by selectively tuning the binding of the buried part of the substrate.

Umbrella sampling QM/MM MD simulations amounting to the nanosecond time scale, at the DFT level, resulted in a kinetic and thermodynamic characterization of the rate-limiting glycosylation step in very good agreement with experimental and computational works published to date. Our calculations confirmed the loose and asynchronous nature of the glycosylation step in α-amylase, which consists of a one-step S_N_2-like nucleophilic substitution of the Asp197 on the anomeric carbon of the buried glucoside of the substrate, preceded by the protonation of the glycosidic oxygen by the Glu233 acid to release the aglycone molecule. In line with the results from other α-glucosidases, the buried glucoside of the substrate binds more favorably in a ^4^*C*_1_ chair conformation that changes to the half-chair ^2^*H*_3_ conformation at the transition state, rapidly converting to the envelope *E*_3_ conformation where the anomeric carbon is most electrophilic. The product state is again characterized by a ^4^*C*_1_ chair conformation. An alternative ^2^*S*_*O*_ skew conformation leads to transition states farther from the half-chair/envelope TS-like conformations and to an Asp197-glucosyl product that can interchange between the ^4^*C*_1_ chair and the more endergonic *B*_3,*O*_ boat conformation.

An analysis of the main interactions involved in the glycosylation step clarified that the glycosidic oxygen of the leaving glucoside unit is particularly responsible for the retention of a bulk water molecule, which promotes enzyme catalysis by lowering the p*K*_a_ of the Glu233 acid. This buried water was confirmed to form a short hydrogen bond with the Glu233 at the transition state, and consequently promote the acid–base reaction with the glycosidic oxygen. The latter reaction is key to lower the Gibbs barrier of the nucleophilic attack of the Asp197 by over 5 kcal·mol^–1^. We observed that the buried water interacts more pronouncedly with the glycosidic oxygen of the substrate, and we finally propose a simple collective variable consisting of a linear combination of distances of the Glu233 and the buried water to the glycosidic oxygen and the distance corresponding to the nucleophilic attack of Asp197 to the anomeric carbon of the buried glucoside, which can be used to identify reactive conformations of enzyme:substrate complexes for α-amylase.

These results are an important contribution for the accurate characterization of enzyme catalysis and dynamics, and they should contribute to improved rational drug design and enzyme engineering campaigns.

## Data and Software Availability

Topology and coordinate files for the solvated α-amylase:5-GA complex are available in the Supporting Information in machine-readable format for Gromacs 2018.3 (SI_gmx-md_inputs.zip) and AMBER18 (SI_amber-md_inputs.zip). Conventional molecular mechanics MD simulations were performed with the Gromacs 2018.3 software, freely available for download at https://manual.gromacs.org/documentation/2018.3. Required files to reproduce the simulations are available in the Supporting Information (SI_gmx-md_inputs.zip). Data collection and analysis were performed with the *trjconv*, *rms*, *mindist*, *distance* and *cluster* modules of Gromacs 2018.3, and the collected data is available in the Supporting Information (rawdata_md.txt). Cremer–Pople puckering angles were calculated from the cp.py python script made available by Hill and Reilly (DOI: 10.1021/ci600492e) and are available in the Supporting Information (cremer-pople_md.txt and rawdata_us-qmmm.txt). QM/MM simulations, as well as DFT calculations in cluster models, were performed with the Orca 4.2.1 software, available free of charge for academic purposes, upon registration, at https://orcaforum.kofo.mpg.de, and the *sander* module of the AMBER 18 package, also available free of charge in AmberTools 18. The AMBER package is a licensed software that can be purchased via the https://ambermd.org Web site for both academic and industrial use. The AmberTools package is available free of charge, upon registration, at https://ambermd.org/GetAmber.php#ambertools. Required files to reproduce the QM/MM simulations are available in the Supporting Information (SI_orca-amber-qmmmm-md_inputs.zip). Data collection and analysis of the QM/MM simulations was performed with the *cpptraj* module available in the AMBER 18 and AmberTools 18 packages, and the collected data is available in the Supporting Information (rawdata_us-qmmm.txt). Free energy analysis of umbrella sampling simulations was run with the WHAM 2.0.11 software, freely available for download at http://membrane.urmc.rochester.edu. In-house python scripts were developed using Python 2.7.18, available free of charge at https://www.python.org/downloads/release/python-2718, and the python modules *pandas* 0.24.2 (https://pypi.org/project/pandas/0.24.2), *numpy* 1.14.0 (https://pypi.org/project/numpy/1.14.0), *scipy* 1.2.3 (https://docs.scipy.org/doc/scipy/release.1.2.3.html), *matplotlib* 2.2.5 (https://matplotlib.org/2.2.3/contents.html). The ONIOM calculations to confirm the choice of the PBE/def2-SVP level of theory for carrying out the QM/MM simulations were conducted in the Gaussian 16 software, which is a licensed software that is available for purchase at https://gaussian.com/pricing. Gaussian input files of the optimized reactant and transition state stationary points are provided in Supporting Information (SI_oniom-qmmm_inputs.zip).

## Supporting Information Available

Details of the Methods section, validation of the choice of the PBE/def2-SVP level of theory to conduct the QM/MM simulations (Figure S1 and Tables S1–S3), density plots of RC and Gibbs energy profiles along the umbrella sampling QM/MM MD simulations (Figures S2 and S3), and auxiliary plots concerning enzyme substrate dynamics (Figures S4–S6) and the rate-limiting glycosylation of α-amylase (Figures S7–S10) (PDF)
Raw data from the microsecond MD runs (TXT)
Raw data from the umbrella sampling QM/MM runs (TXT)
Raw data from the microsecond MD runs on the Cremer–Pople puckering angles (TXT)
Input files for energy minimization and conventional MD runs (ZIP)
AMBER input files for preparation of models for QM/MM simulations (ZIP)
Input files for QM/MM energy minimization, equilibration, and steered MD runs (ZIP)
Gaussian input files for the stationary points of the ONIOM QM/MM calculations (ZIP)
Orca input files required to carry out the DFT calculations in cluster models (ZIP)

## References

[ref1] Hanoian P.; Liu C. T.; Hammes-Schiffer S.; Benkovic S. Perspectives on Electrostatics and Conformational Motions in Enzyme Catalysis. Acc. Chem. Res. 2015, 48, 482–489. 10.1021/ar500390e. 25565178 PMC4334233

[ref2] Callender R.; Dyer R. B. The Dynamical Nature of Enzymatic Catalysis. Acc. Chem. Res. 2015, 48, 407–413. 10.1021/ar5002928. 25539144 PMC4333057

[ref3] Warshel A.; Bora R. P. Perspective: Defining and quantifying the role of dynamics in enzyme catalysis. J. Chem. Phys. 2016, 144, 180901 10.1063/1.4947037. 27179464 PMC4866948

[ref4] Sousa S. F.; Ribeiro A. J. M.; Neves R. P. P.; Bras N. F.; Cerqueira N. M. F. S. A.; Fernandes P. A.; Ramos M. J. Application of quantum mechanics/molecular mechanics methods in the study of enzymatic reaction mechanisms. WIREs Comput. Mol. Sci. 2017, 7, e1281 10.1002/wcms.1281.

[ref5] Brunk E.; Rothlisberger U. Mixed Quantum Mechanical/Molecular Mechanical Molecular Dynamics Simulations of Biological Systems in Ground and Electronically Excited States. Chem. Rev. 2015, 115, 6217–6263. 10.1021/cr500628b. 25880693

[ref6] Himo F. Recent Trends in Quantum Chemical Modeling of Enzymatic Reactions. J. Am. Chem. Soc. 2017, 139, 6780–6786. 10.1021/jacs.7b02671. 28493715

[ref7] Yang Z. Y.; Mehmood R.; Wang M. Y.; Qi H. W.; Steeves A. H.; Kulik H. J. Revealing quantum mechanical effects in enzyme catalysis with large-scale electronic structure simulation. React. Chem. Eng. 2019, 4, 298–315. 10.1039/C8RE00213D. 31572618 PMC6768422

[ref8] Aqvist J.; Warshel A. Computer-Simulation of the Initial Proton-Transfer Step in Human Carbonic Anhydrase-I. J. Mol. Biol. 1992, 224, 7–14. 10.1016/0022-2836(92)90572-2. 1312606

[ref9] Warshel A. Energetics of Enzyme Catalysis. Proc. Natl. Acad. Sci. U. S. A. 1978, 75, 5250–5254. 10.1073/pnas.75.11.5250. 281676 PMC392938

[ref10] Warshel A.; Sharma P. K.; Kato M.; Xiang Y.; Liu H. B.; Olsson M. H. M. Electrostatic basis for enzyme catalysis. Chem. Rev. 2006, 106, 3210–3235. 10.1021/cr0503106. 16895325

[ref11] Warshel A. Electrostatic origin of the catalytic power of enzymes and the role of preorganized active sites. J. Biol. Chem. 1998, 273, 27035–27038. 10.1074/jbc.273.42.27035. 9765214

[ref12] Stevens D. R.; Hammes-Schiffer S. Exploring the Role of the Third Active Site Metal Ion in DNA Polymerase eta with QM/MM Free Energy Simulations. J. Am. Chem. Soc. 2018, 140, 8965–8969. 10.1021/jacs.8b05177. 29932331 PMC6399739

[ref13] Allec S. I.; Sun Y. J.; Sun J. A.; Chang C. E. A.; Wong B. M. Heterogeneous CPU plus GPU-Enabled Simulations for DFTB Molecular Dynamics of Large Chemical and Biological Systems. J. Chem. Theor Comp 2019, 15, 2807–2815. 10.1021/acs.jctc.8b01239. PMC8285072 30916958

[ref14] Vaissier Welborn V.; Head-Gordon T. Computational Design of Synthetic Enzymes. Chem. Rev. 2019, 119, 6613–6630. 10.1021/acs.chemrev.8b00399. 30277066

[ref15] Korendovych I. V. Rational and Semirational Protein Design. In Protein Engineering: Methods and Protocols; Bornscheuer U. T., Höhne M., Eds.; Springer New York: New York, NY, 2018; pp 15–23. 10.1007/978-1-4939-7366-8_2 PMC5912912 29086301

[ref16] Acebes S.; Fernandez-Fueyo E.; Monza E.; Lucas M. F.; Almendral D.; Ruiz-Duenas F. J.; Lund H.; Martinez A. T.; Guallar V. Rational Enzyme Engineering Through Biophysical and Biochemical Modeling. ACS Catal. 2016, 6, 1624–1629. 10.1021/acscatal.6b00028.

[ref17] Socan J.; Isaksen G. V.; Brandsdal B. O.; Aqvist J. Towards Rational Computational Engineering of Psychrophilic Enzymes. Sci. Rep-Uk 2019, 9, 19147 10.1038/s41598-019-55697-4. PMC6915740 31844096

[ref18] Sousa J. P. M.; Ferreira P.; Neves R. P. P.; Ramos M. J.; Fernandes P. A. The bacterial 4S pathway – an economical alternative for crude oil desulphurization that reduces CO2 emissions. Green Chem. 2020, 22, 7604–7621. 10.1039/D0GC02055A.

[ref19] Wolfenden R.; Ridgway C.; Young G. Spontaneous hydrolysis of ionized phosphate monoesters and diesters and the proficiencies of phosphatases and phosphodiesterases as catalysts. J. Am. Chem. Soc. 1998, 120, 833–834. 10.1021/ja9733604.

[ref20] Wolfenden R.; Lu X. D.; Young G. Spontaneous hydrolysis of glycosides. J. Am. Chem. Soc. 1998, 120, 6814–6815. 10.1021/ja9813055.

[ref21] Nishimura Y. Gem-diamine 1-N-iminosugars and related iminosugars, candidate of therapeutic agents for tumor metastasis. Curr. Top Med. Chem. 2003, 3, 575–591. 10.2174/1568026033452492. 12570867

[ref22] Stütz A. Iminosugars as Glycosidase Inhibitors: Nojirimycin and Beyond; Wiley-VCH: Weinheim, Germany, and New York, 1999.

[ref23] Deng Z. M.; Yang H. Q.; Shin H. D.; Li J. H.; Liu L. Structure-based rational design and introduction of arginines on the surface of an alkaline α-amylase from Alkalimonas amylolytica for improved thermostability. Appl. Microbiol. Biotechnol. 2014, 98, 8937–8945. 10.1007/s00253-014-5790-8. 24816623

[ref24] D’Amico S.; Gerday C.; Feller G. Temperature adaptation of proteins: Engineering mesophilic-like activity and stability in a cold-adapted α-amylase. J. Mol. Biol. 2003, 332, 981–988. 10.1016/j.jmb.2003.07.014. 14499602

[ref25] Jayaraj S.; Suresh S.; Kadeppagari R. K. Amylase inhibitors and their biomedical applications. Starch-Starke 2013, 65, 535–542. 10.1002/star.201200194.

[ref26] Oliveira H.; Fernandes A.; Bras N. F.; Mateus N.; de Freitas V.; Fernandes I. Anthocyanins as Antidiabetic Agents-In Vitro and In Silico Approaches of Preventive and Therapeutic Effects. Molecules 2020, 25, 3813 10.3390/molecules25173813. 32825758 PMC7504281

[ref27] Antranikian G. Microbial degradation of starch. In Microbial degradation of natural products; VCH Verlagsgesellschaft mbH: Weinheim, Germany, 1992; pp 27–56.

[ref28] Mehta D.; Satyanarayana T. Bacterial and Archaeal alpha-Amylases: Diversity and Amelioration of the Desirable Characteristics for Industrial Applications. Front Microbiol 2016, 7, 1129 10.3389/fmicb.2016.01129. 27516755 PMC4963412

[ref29] Pinto G. P.; Brás N. F.; Perez M. A. S.; Fernandes P. A.; Russo N.; Ramos M. J.; Toscano M. Establishing the Catalytic Mechanism of Human Pancreatic α-Amylase with QM/MM Methods. J. Chem. Theor Comp 2015, 11, 2508–2516. 10.1021/acs.jctc.5b00222. 26575550

[ref30] Koshland D. E. Stereochemistry and the Mechanism of Enzymatic Reactions. Biol. Rev. 1953, 28, 416–436. 10.1111/j.1469-185X.1953.tb01386.x.

[ref31] Rydberg E. H.; Li C. M.; Maurus R.; Overall C. M.; Brayer G. D.; Withers S. G. Mechanistic analyses of catalysis in human pancreatic alpha-amylase: Detailed kinetic and structural studies of mutants of three conserved carboxylic acids. Biochemistry 2002, 41, 4492–4502. 10.1021/bi011821z. 11914097

[ref32] Pauling L. Molecular Architecture and Biological Reactions. Chem. Eng. News 1946, 24, 1375–1377. 10.1021/cen-v024n010.p1375.

[ref33] Bras N. F.; Fernandes P. A.; Ramos M. J. QM/MM Studies on the beta-Galactosidase Catalytic Mechanism: Hydrolysis and Transglycosylation Reactions. J. Chem. Theor Comp 2010, 6, 421–433. 10.1021/ct900530f. 26617299

[ref34] Ardevol A.; Rovira C. Reaction Mechanisms in Carbohydrate-Active Enzymes: Glycoside Hydrolases and Glycosyltransferases. Insights from ab Initio Quantum Mechanics/Molecular Mechanics Dynamic Simulations. J. Am. Chem. Soc. 2015, 137, 7528–7547. 10.1021/jacs.5b01156. 25970019

[ref35] Santos-Martins D.; Calixto A. R.; Fernandes P. A.; Ramos M. J. A Buried Water Molecule Influences Reactivity in a α-Amylase on a Subnanosecond Time Scale. ACS Catal. 2018, 8, 4055–4063. 10.1021/acscatal.7b04400.

[ref36] Ribeiro A. J. M.; Santos-Martins D.; Russo N.; Ramos M. J.; Fernandes P. A. Enzymatic Flexibility and Reaction Rate: A QM/MM Study of HIV-1 Protease. ACS Catal. 2015, 5, 5617–5626. 10.1021/acscatal.5b00759.

[ref37] Kosugi T.; Hayashi S. Crucial Role of Protein Flexibility in Formation of a Stable Reaction Transition State in an α-Amylase Catalysis. J. Am. Chem. Soc. 2012, 134, 7045–7055. 10.1021/ja212117m. 22468622

[ref38] Nerinckx W.; Desmet T.; Piens K.; Claeyssens M. An elaboration on the syn–anti proton donor concept of glycoside hydrolases: electrostatic stabilisation of the transition state as a general strategy. FEBS Lett. 2005, 579, 302–312. 10.1016/j.febslet.2004.12.021. 15642336

[ref39] Brayer G. D.; Sidhu G.; Maurus R.; Rydberg E. H.; Braun C.; Wang Y. L.; Nguyen N. T.; Overall C. H.; Withers S. G. Subsite mapping of the human pancreatic alpha-amylase active site through structural, kinetic, and mutagenesis techniques. Biochemistry 2000, 39, 4778–4791. 10.1021/bi9921182. 10769135

[ref40] Abraham M. J.; Murtola T.; Schulz R.; Páll S.; Smith J. C.; Hess B.; Lindahl E. GROMACS: High performance molecular simulations through multi-level parallelism from laptops to supercomputers. SoftwareX 2015, 1–2, 19–25. 10.1016/j.softx.2015.06.001.

[ref41] Case D. A.; Ben-Shalom I. Y.; Brozell S. R.; Cerutti D. S.; Cheatham T. E. III; Cruzeiro V. W. D.; Darden T. A.; Duke R. E.; Ghoreishi D.; Gilson M. K.; Gohlke H.; Goetz A. W.; Greene D.; Harris R.; Homeyer N.; Izadi S.; Kovalenko A.; Kurtzman T.; Lee T. S.; LeGrand S.; Li P.; Lin C.; Liu J.; Luchko T.; Luo R.; Mermelstein D. J.; Merz K. M.; Miao Y.; Monard G.; Nguyen C.; Nguyen H.; Omelyan I.; Onufriev A.; Pan F.; Qi R.; Roe D. R.; Roitberg A.; Sagui C.; Schott-Verdugo S.; Shen J.; Simmerling C. L.; Smith J.; Salomon-Ferrer R.; Swails J.; Walker R. C.; Wang J.; Wei H.; Wolf R. M.; Wu X.; Xiao L.; York D. M.; Kollman P. A. Amber 18; University of California: San Francisco, CA, 2018.

[ref42] Hess B. P-LINCS: A parallel linear constraint solver for molecular simulation. J. Chem. Theor Comp 2008, 4, 116–122. 10.1021/ct700200b. 26619985

[ref43] Miyamoto S.; Kollman P. A. SETTLE: An Analytical Version of the Shake and Rattle Algorithm for Rigid Water Models. J. Comput. Chem. 1992, 13, 952–962. 10.1002/jcc.540130805.

[ref44] Ewald P. P. The calculation of optical and electrostatic grid potential. Ann. Phys-Berlin 1921, 369, 253–287. 10.1002/andp.19213690304.

[ref45] Berendsen H. J. C.; Postma J. P. M.; Vangunsteren W. F.; Dinola A.; Haak J. R. Molecular-Dynamics with Coupling to an External Bath. J. Chem. Phys. 1984, 81, 3684–3690. 10.1063/1.448118.

[ref46] Bussi G.; Donadio D.; Parrinello M. Canonical sampling through velocity rescaling. J. Chem. Phys. 2007, 126, 014101 10.1063/1.2408420. 17212484

[ref47] Parrinello M.; Rahman A. Polymorphic Transitions in Single-Crystals - a New Molecular-Dynamics Method. J. Appl. Phys. 1981, 52, 7182–7190. 10.1063/1.328693.

[ref48] Daura X.; Gademann K.; Jaun B.; Seebach D.; van Gunsteren W. F.; Mark A. E. Peptide folding: When simulation meets experiment. Angew. Chem. Int. Edit 1999, 38, 236–240. 10.1002/(SICI)1521-3773(19990115)38:1/2<236::AID-ANIE236>3.0.CO;2-M.

[ref49] Neese F. Software update: the ORCA program system, version 4.0. WIREs Comput. Mol. Sci. 2018, 8, e1327 10.1002/wcms.1327.

[ref50] Neese F. The ORCA program system. WIREs Comput. Mol. Sci. 2012, 2, 73–78. 10.1002/wcms.81.

[ref51] Perdew J. P.; Burke K.; Ernzerhof M. Generalized gradient approximation made simple. Phys. Rev. Lett. 1996, 77, 3865–3868. 10.1103/PhysRevLett.77.3865. 10062328

[ref52] Biarnés X.; Ardèvol A.; Iglesias-Fernández J.; Planas A.; Rovira C. Catalytic Itinerary in 1,3–1,4-β-Glucanase Unraveled by QM/MM Metadynamics. Charge Is Not Yet Fully Developed at the Oxocarbenium Ion-like Transition State. J. Am. Chem. Soc. 2011, 133, 20301–20309. 10.1021/ja207113e. 22044419

[ref53] Alonso-Gil S.; Coines J.; Andre I.; Rovira C. Conformational Itinerary of Sucrose During Hydrolysis by Retaining Amylosucrase. Front Chem. 2019, 7, 269 10.3389/fchem.2019.00269. 31114783 PMC6502901

[ref54] Cuxart I.; Coines J.; Esquivias O.; Faijes M.; Planas A.; Biarnés X.; Rovira C. Enzymatic Hydrolysis of Human Milk Oligosaccharides. The Molecular Mechanism of Bifidobacterium Bifidum Lacto-N-biosidase. ACS Catal. 2022, 12, 4737–4743. 10.1021/acscatal.2c00309. 35465242 PMC9016705

[ref55] Teze D.; Coines J.; Fredslund F.; Dubey K. D.; Bidart G. N.; Adams P. D.; Dueber J. E.; Svensson B.; Rovira C.; Welner D. H. O-/N-/S-Specificity in Glycosyltransferase Catalysis: From Mechanistic Understanding to Engineering. ACS Catal. 2021, 11, 1810–1815. 10.1021/acscatal.0c04171.

[ref56] Csonka G. I.; French A. D.; Johnson G. P.; Stortz C. A. Evaluation of Density Functionals and Basis Sets for Carbohydrates. J. Chem. Theor Comp 2009, 5, 679–692. 10.1021/ct8004479. 26609572

[ref57] Marianski M.; Supady A.; Ingram T.; Schneider M.; Baldauf C. Assessing the Accuracy of Across-the-Scale Methods for Predicting Carbohydrate Conformational Energies for the Examples of Glucose and α-Maltose. J. Chem. Theor Comp 2016, 12, 6157–6168. 10.1021/acs.jctc.6b00876. 27951668

[ref58] Pereira A. T.; Ribeiro A. J. M.; Fernandes P. A.; Ramos M. J. Benchmarking of density functionals for the kinetics and thermodynamics of the hydrolysis of glycosidic bonds catalyzed by glycosidases. Int. J. Quantum Chem. 2017, 117, e25409 10.1002/qua.25409.

[ref59] Dohn A. O. Multiscale electrostatic embedding simulations for modeling structure and dynamics of molecules in solution: A tutorial review. Int. J. Quantum Chem. 2020, 120, e26343 10.1002/qua.26343.

[ref60] Cruzeiro V. W. D.; Manathunga M.; Merz K. M.; Gotz A. W. Open-Source Multi-GPU-Accelerated QM/MM Simulations with AMBER and QUICK. J. Chem. Inf Model 2021, 61, 2109–2115. 10.1021/acs.jcim.1c00169. 33913331

[ref61] Teixeira M. H.; Curtolo F.; Camilo S. R. G.; Field M. J.; Zheng P.; Li H. B.; Arantes G. M. Modeling the Hydrolysis of Iron-Sulfur Clusters. J. Chem. Inf Model 2020, 60, 653–660. 10.1021/acs.jcim.9b00881. 31790241

[ref62] Frisch M. J.; Trucks G. W.; Schlegel H. B.; Scuseria G. E.; Robb M. A.; Cheeseman J. R.; Scalmani G.; Barone V.; Petersson G. A.; Nakatsuji H.; Li X.; Caricato M.; Marenich A. V.; Bloino J.; Janesko B. G.; Gomperts R.; Mennucci B.; Hratchian H. P.; Ortiz J. V.; Izmaylov A. F.; Sonnenberg J. L.; Williams; Ding F.; Lipparini F.; Egidi F.; Goings J.; Peng B.; Petrone A.; Henderson T.; Ranasinghe D.; Zakrzewski V. G.; Gao J.; Rega N.; Zheng G.; Liang W.; Hada M.; Ehara M.; Toyota K.; Fukuda R.; Hasegawa J.; Ishida M.; Nakajima T.; Honda Y.; Kitao O.; Nakai H.; Vreven T.; Throssell K.; Montgomery J. A. Jr.; Peralta J. E.; Ogliaro F.; Bearpark M. J.; Heyd J. J.; Brothers E. N.; Kudin K. N.; Staroverov V. N.; Keith T. A.; Kobayashi R.; Normand J.; Raghavachari K.; Rendell A. P.; Burant J. C.; Iyengar S. S.; Tomasi J.; Cossi M.; Millam J. M.; Klene M.; Adamo C.; Cammi R.; Ochterski J. W.; Martin R. L.; Morokuma K.; Farkas O.; Foresman J. B.; Fox D. J. Gaussian 16, Rev. B01; Wallingford, CT, 2016.

[ref63] Crespo A.; Marti M. A.; Estrin D. A.; Roitberg A. E. Multiple-steering QM-MM calculation of the free energy profile in chorismate mutase. J. Am. Chem. Soc. 2005, 127, 6940–6941. 10.1021/ja0452830. 15884923

[ref64] Grossfield A. WHAM: the weighted histogram analysis method, ver. 2.0.9; Rochester, NY, 2013.

[ref65] Biarnés X.; Ardevol A.; Planas A.; Rovira C.; Laio A.; Parrinello M. The conformational free energy landscape of beta-D-glucopyranose. Implications for substrate preactivation in beta-glucoside hydrolases. J. Am. Chem. Soc. 2007, 129, 10686 10.1021/ja068411o. 17696342

[ref66] Taylor E. J.; Goyal A.; Guerreiro C. I. P. D.; Prates J. A. M.; Money V. A.; Ferry N.; Morland C.; Planas A.; Macdonald J. A.; Stick R. V.; Gilbert H. J.; Fontes C. M. G. A.; Davies G. J. How family 26 glycoside hydrolases orchestrate catalysis on different polysaccharides - Structure and activity of a clostridium thermocellum lichenase, CtLic26A. J. Biol. Chem. 2005, 280, 32761–32767. 10.1074/jbc.M506580200. 15987675

[ref67] Davies G. J.; Planas A.; Rovira C. Conformational Analyses of the Reaction Coordinate of Glycosidases. Acc. Chem. Res. 2012, 45, 308–316. 10.1021/ar2001765. 21923088

[ref68] Mayes H. B.; Broadbelt L. J.; Beckham G. T. How Sugars Pucker: Electronic Structure Calculations Map the Kinetic Landscape of Five Biologically Paramount Monosaccharides and Their Implications for Enzymatic Catalysis. J. Am. Chem. Soc. 2014, 136, 1008–1022. 10.1021/ja410264d. 24368073

[ref69] Calixto A. R.; Ramos M. J.; Fernandes P. A. Conformational diversity induces nanosecond-timescale chemical disorder in the HIV-1 protease reaction pathway. Chem. Sci. 2019, 10, 7212–7221. 10.1039/C9SC01464K. 31588289 PMC6677113

