# Supplementary material for: Role of Enzyme and Active Site Conformational Dynamics in the Catalysis by α-Amylase Explored with QM/MM Molecular Dynamics
Source: J Chem Inf Model. 2022 Jul 26;62(15):3638–50. doi: 10.1021/acs.jcim.2c00691 (PMC9778734; doi:10.1021/acs.jcim.2c00691)
Supplement: Supplementary file 1 — ci2c00691_si_001.pdf [file ci2c00691_si_001.pdf]

SUPPORTING INFORMATION

The role of enzyme and active site conformational dynamics in the catalysis by α-amylase explored with QM/MM molecular dynamics

Rui P. P. Neves, Pedro A. Fernandes, Maria J. Ramos*

LAQV@REQUIMTE, Departamento de Química e Bioquímica, Faculdade de Ciências, Universidade do Porto, Rua do Campo Alegre s/n, 4169-007 Porto, Portugal

*e-mail: rui.neves@fc.up.pt

Details on the methods section

Parameterizing enzyme:substrate model.

The AMBER 18 package[1] was used to run all calculations. Each of the 4 models was subjected to a prior geometry optimization at the molecular mechanics level, in four stages: (1) energy minimization with all solute atoms restrained with harmonic potential force constants of 50 kcal·mol^{-1}·Å^{-2}; (2) energy minimization with every non-hydrogen atom restrained with 50 kcal·mol^{-1}·Å^{-2} harmonic force constants; (3) energy minimization with the backbone of the protein solute restrained with 50 kcal·mol^{-1}·Å^{-2} harmonic force constants; and (4) energy minimization without any external constraints. The energy minimization protocol followed the combined steepest descent/conjugate gradient method. Non-bonded interactions were treated explicitly within 10 Å of each particle; beyond that, Coulomb interactions were treated with the particle-mesh Ewald scheme and Lennard-Jones interactions were truncated. The final optimized coordinates were used for subsequent microsecond conventional MD simulations and quantum mechanics/molecular mechanics (QM/MM) calculations.

Microsecond conventional MD.

An initial annealing simulation was run for 100 ps, with constant *N* and *V*, up to a reference temperature of 310 K, followed by a 2 ns equilibration of the density of the solvent in an *NPT* ensemble. An integration timestep of 2 fs was used, using the LINCS algorithm[2, 3] to constrain all hydrogen covalent bonds, and a cut-off of 10 Å was used to define explicit particle-particle electrostatic and Lennard-Jones interactions. Electrostatic interactions beyond 10 Å were treated with the particle-mesh Ewald summation method,[4] using a cubic interpolation grid and a 1.2 Å Fourier-spacing. The pressure of the system was set to 1 bar, coupled with a 2 ps relaxation time isotropic Berendsen barostat,[5] and a reference temperature of 310 K was set for the solute and solvent, which were coupled independently with a 0.1 ps relaxation time velocity-rescaling thermostat.[6]

Validation of the PBE/def2-SVP:AMBER level to describe the glycosylation step

We evaluated the performance of the PBE/def2-SVP combination to carry out the QM calculations to characterize the glycosylation step of α-amylase.

To do so, we took the QM/MM optimized reactant state of each of the three conformations (A, B and C) that we chose to carry out our study, which were available in the Supporting Information of the work of Santos-Martins et al. (ACS Catalysis, 2018). We have then repeated the glycosylation step at the PBE/def2-SVP:AMBER level of theory, using the ONIOM methodology and the electrostatic embedding scheme, as implemented in Gaussian 16. The link-atom approach was used to fill the valences of the capped atoms included in the QM layer. The glycosylation step was studied using the same reaction coordinate (RC) as in our umbrella sampling simulations: $d_{glyc} - d_{acid}$.

Our calculations indicated that overall the glycosylation reaction is in agreement with that previously characterized by Pinto et al. and Santos-Martins et al. at the ONIOM(B3LYP/6-31G*:AMBER) level,[7, 8] as can be confirmed in Table S1. Upon comparison of the distances that were previously defined to be more relevant for the glycosylation step to occur, most differences between geometry optimizations at the ONIOM(PBE/def2-SVP:AMBER) and ONIOM(B3LYP/6-31G*:AMBER) are below 0.1 Å.

Table S1. Most relevant distances analyzed by Santos-Martins et al. to characterize reactive enzyme:substrate complexes of α-amylase, determined at ONIOM(PBE/def2-SVP:AMBER) level. The reference results were taken to be those by Santos-Martins et al. obtained at the ONIOM(B3LYP/6-31G*:AMBER) level and are indicated in superscript (the red values correspond to those of an alternative TS calculated with the $d_{glyc} - d_{acid}$ as reaction coordinate). The single imaginary frequencies, verified to correspond to the transition state of the reaction, are also included.

Conformation	d_{wat} (R)	d_{acid} (R)	d_{nuc} (R)	d_{wat} (TS)	d_{acid} (TS)	d_{nuc} (TS)	imaginary frequency (cm^{-1})
			(all distances in Å)				
A	3.22 $^{3.29}$	2.77 $^{2.79}$	3.42 $^{3.44}$	3.11 $^{2.10 / 3.03}$	1.28 $^{0.98 / 1.52}$	2.61 $^{2.70 / 2.48}$	i300.6 $^{i150.5 / i122.6}$
B	2.57 $^{2.92}$	1.60 $^{1.89}$	3.17 $^{3.24}$	2.07 $^{2.08}$	1.04 $^{1.10}$	2.74 $^{2.90}$	i49.4 $^{i153.0}$
C	2.94 $^{2.89}$	2.50 $^{2.48}$	3.52 $^{3.47}$	2.06 $^{2.13}$	1.05 $^{1.10}$	2.60 $^{2.70}$	i31.3 $^{i209.2}$

Only the TS of conformation A, which corresponds to the highest energy barrier calculated for the glycosylation reaction, differs more from the determined by Santos-Martins et al. An analysis of the displacement vectors of the imaginary frequency characteristic of each TS (Figure S1), indicates that the proton transfer from the Glu233 to the glycosidic oxygen in the cleaved glycosidic bond is the dominant vibration in this conformation. This is also in agreement with the significantly higher value of the imaginary frequency of the TS of conformation A

(i300.6 vs. i31.3/i49.4 cm^{-1} in conformations B or C), whereas for conformations B and C, largest displacement vectors are centered in the heavy atoms of the glucoside ring binding the nucleophilic Asp197.

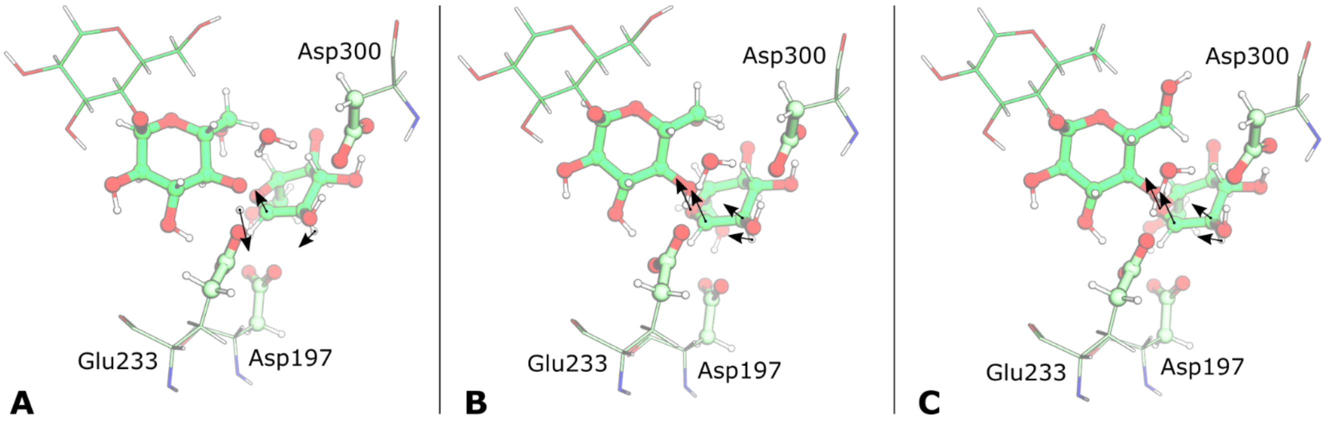

Figure S1. Schematic representation of the largest atom-centered vectors for the imaginary frequency corresponding to the transition state of conformations **A**, **B** and **C**, calculated for the glycosylation reaction, at the ONIOM(PBE/def2-SVP:AMBER) level.

Since a different coordinate was previously used to study the reaction (d_{acid}), we repeated the linear transit scan with our defined reaction coordinate ($d_{glyc} - d_{acid}$) at the ONIOM(B3LYP/6-31G*:AMBER) level, for the conformation A to assess if the observed differences stemmed from the different reaction coordinate used, instead of the density functional, which we confirmed to be the case (refer to red values indicated in Table S1).

We then ran single-point energy calculations at the ONIOM(PBE/def2-TZVPP:AMBER) level, to assess the effect of a larger basis set in the energy barriers of the glycosylation step. Except for conformation A, which corresponded to the highest energy barrier, energy barriers varied below 1 kcal·mol^{-1}, suggesting that similar results might be expected from the combination of PBE with either smaller or larger basis sets. Since previous studies indicated that PBE underestimates the energy barrier of the glycosylation reaction,[9] we tested the M06-2X/6-311++G(2d,2p) level, previously used by Santos-Martins et al. in their multi-PES QM/MM study of the glycosylation of α-amylase, and the PBE0/aug-cc-pVTZ level, identified in a previous benchmark on the glycosylation reaction by Pereira et al. to accurately determine reaction barriers and energy for the glycosylation reaction. The results are compiled in Table S2. Since the computational cost of the ONIOM(PBE0/aug-cc-pVTZ:AMBER) calculations was verified to be very high, we also performed the ONIOM calculations with a large basis set reported to be more stable for DFT calculations (def2-TZVPP). Finally, we performed DFT calculations for the QM layer of each conformation with increasing Ahlrichs basis sets (def2-SVP, def2-TZVP and def2-TZVPP) and the aug-cc-pVTZ basis sets to assess energy differences between these basis sets (Table S3), using the Orca 4.2.1 software.[10] All the valences of the capped atoms of the QM layer were filled with hydrogen atoms.

Table S2. Energy barriers for the glycosylation step calculated from ONIOM calculations ran on geometries determined at the ONIOM(PBE/def2-SVP:AMBER) employing different combinations of density functionals and basis sets: PBE/def2-SVP, M06-2X/6-311++G(2d,2p) and PBE0/def2-TZVPP. The energy barriers determined at the ONIOM(M06-2X/6-311++G(2d,2p):AMBER//B3LYP/6-31G*:AMBER) level, as by Santos-Martins et al., are indicated in superscript. All values are in kcal·mol^{-1}.

**Conformation**	$\Delta E^{‡}$ (PBE/def2-SVP:AMBER)	$\Delta E^{‡}$ (PBE/def2-TZVPP:AMBER)	$\Delta E^{‡}$ (M06-2X/6-311++G(2d,2p):AMBER)	$\Delta E^{‡}$ (PBE0/def2-TZVPP:AMBER)	$\Delta E^{‡}$ (PBE0/aug-cc-pVTZ:AMBER)
	geometry optimization at ONIOM(PBE/def2-SVP:AMBER) level				
A	28.5	25.7	36.0 $^{37.2}$	33.8	33.6
B	8.3	7.6	12.8 $^{11.3}$	11.8	12.1
C	14.1	14.8	22.5 $^{21.3}$	21.3	21.5

Table S3. Energy barriers for the glycosylation step calculated from DFT calculations with PBE0, ran on the QM layer of geometries determined at the ONIOM(PBE/def2-SVP:AMBER), with varying basis sets: def2-SVP, def2-TZVP, def2-TZVPP and aug-cc-pVTZ. All values are in kcal·mol^{-1}.

**Conformation**	$\Delta E^{‡}$ (PBE0/def2-SVP)	$\Delta E^{‡}$ (PBE0/def2-TZVP)	$\Delta E^{‡}$ (PBE0/def2-TZVPP)	$\Delta E^{‡}$ (PBE0/aug-cc-pVTZ)
A	37.9	34.6	34.9	35.5
B	11.1	9.9	10.1	10.4
C	19.7	19.5	19.7	19.4

We confirmed that energy barriers determined at the PBE level are lower than those determined at the M06-2X and PBE0. Both M06-2X and PBE0 provide similar results, in particular for the conformations where lower energy barriers are observed (B and C).

After running single-point energy calculations with both small (def2-SVP) and large (def2-TZVPP) basis sets, we observed that energy barriers do not vary pronouncedly with the basis set size (they vary around 1 kcal·mol^{-1} for either PBE or PBE0). In addition, the correction from the PBE0/def2-TZVPP to the PBE0-aug-cc-pVTZ is generally below 0.5 kcal·mol^{-1}. In particular, the combination of PBE and def2-SVP leads to energy barriers closer to those calculated at the M06-2X/6-311++G(2d,2p) and PBE0/aug-cc-pVTZ levels, than when def2-TZVPP is employed.

While any density functional accurately ranked the glycosylation reaction, from higher (conformation A) to lower (conformation B) energy barrier, energy differences between density functionals are not constant (they span from 4.5 to 8.4 kcal·mol^{-1} with M06-2X/6-311++G(2d,2p) or from 3.8 to 7.0 kcal·mol^{-1} with PBE0/aug-cc-pVTZ),

although our results seem to indicate that higher energy barriers lead to larger energy differences between density functionals, which would indicate that the PBE/def2-SVP level can adequately distinguish the more favourable pathways to carry out the glycosylation reaction.

Together with the available body of work using PBE to study this family of enzymes,[11-14] we deem the PBE/def2-SVP:AMBER level of theory adequate to study the geometry changes characteristic of the glycosylation reaction, as well as the energetics of the glycosylation step of α-amylase.

Umbrella sampling QM/MM MD.

All QM/MM calculations were carried out with the AMBER 18 package, with high-level calculations performed externally by the Orca 4.1.2 software, and using the Born-Oppenheimer approximation.[10, 15] The energy minimization protocol was carried out with 50 steps of steepest descent search followed by a conjugate gradient search up to 200 cycles of minimization, followed by a short 2 ps MD simulation in the NPT ensemble (1 bar and 310 K) in a periodic box. The Langevin thermostat with a collision frequency of 1 ps^{-1}, and the Monte Carlo barostat with a relaxation time of 2 ps were employed. Non-bonded interactions were treated explicitly within 9 Å of each particle; beyond that, Coulomb interactions were treated with the particle-mesh Ewald scheme and Lennard-Jones interactions were truncated. An integration step of 1 fs was used to integrate motion equations.

For each of the 4 models selected for QM/MM MD simulations, a boxplot distribution of the RC was performed at the reactant and product states, to set up the upper and lower limit of the RC to be tested with umbrella sampling MD simulations. The upper and lower limits were set using the largest amplitudes attainable from either the first and third quartiles of the distribution for both the reactant and product state. A harmonic potential with a length of 2 Å and a force constant of 100 $kcal{\cdot}mol^{-1}{\cdot}Å^{-2}$ was centered every 0.10 Å along the amplitude defined from the boxplot distributions for each reaction coordinates. Initial coordinates for umbrella sampling MD simulations were taken to be the ones from the steered MD trajectory that were the closest to the coordinate where the harmonic potential was centered. Every umbrella sampling MD simulation was run for 10 ps (resulting in a total of 450-500 ps per QM/MM model), writing RC values every 5 fs. The simulations were processed with the weighted-histogram analysis method as implemented in the WHAM software, by Grossman et al, using 100 bootstrap datasets and a 10^{-4} tolerance threshold along a number of bins that was twice the number of independent simulated windows. Time-dependent block analysis was carried out in the direct and reverse timeseries in order to evaluate the statistical convergence of the resulting potential of mean force (PMF) plots for the glycosidic cleavage step. Simulations with an average RC distribution (μ) 0.20 Å higher than the reference minimum (resulting in poor histogram overlap) were repeated with a harmonic potential with a harmonic force constant of 200 $kcal{\cdot}mol^{-1}{\cdot}Å^{-2}$. The 0.20 Å threshold was defined as *ca.* twice that of the standard deviation (2σ) of the distribution of the RC along every simulation (~0.1 Å), considering that this range should account for about 95% of the simulated population if a Gaussian distribution was assumed.

X-ray

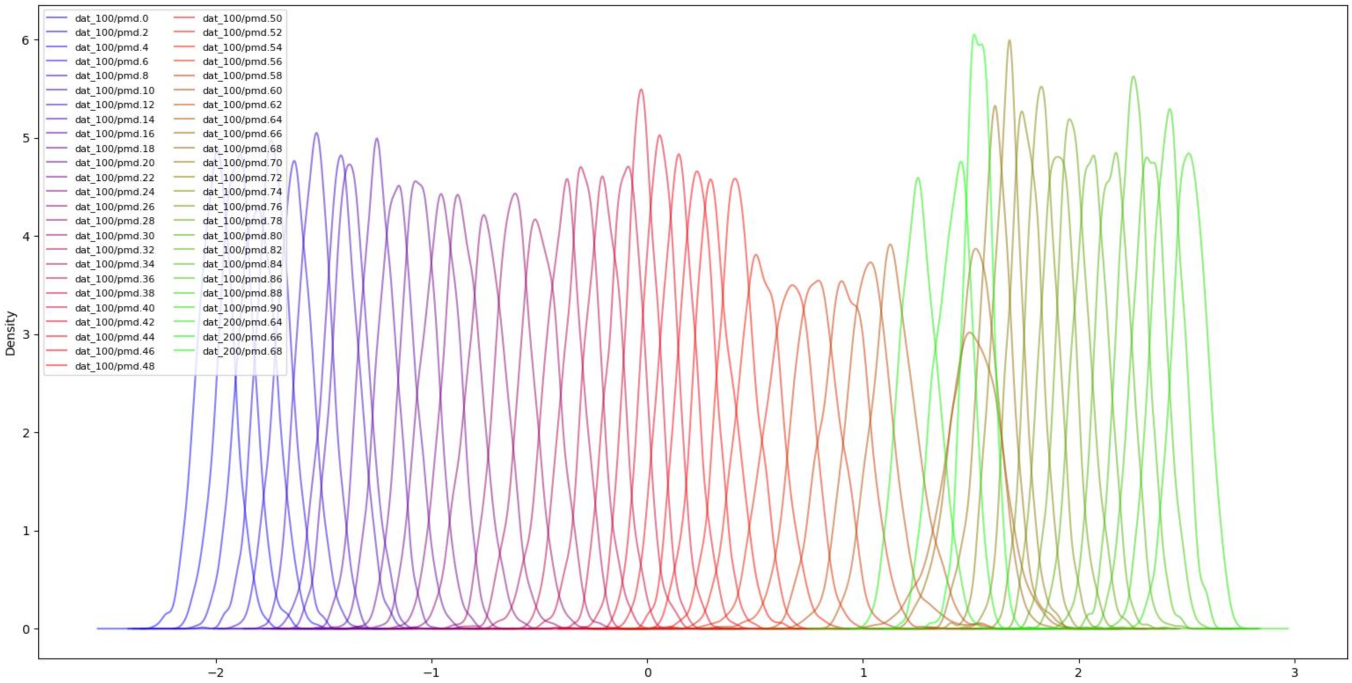

dat_100/pmd.0
dat_100/pmd.2
dat_100/pmd.4
dat_100/pmd.6
dat_100/pmd.8
dat_100/pmd.10
dat_100/pmd.12
dat_100/pmd.14
dat_100/pmd.16
dat_100/pmd.18
dat_100/pmd.20
dat_100/pmd.22
dat_100/pmd.24
dat_100/pmd.26
dat_100/pmd.28
dat_100/pmd.30
dat_100/pmd.32
dat_100/pmd.34
dat_100/pmd.36
dat_100/pmd.38
dat_100/pmd.40
dat_100/pmd.42
dat_100/pmd.44
dat_100/pmd.46
dat_100/pmd.48
dat_100/pmd.50
dat_100/pmd.52
dat_100/pmd.54
dat_100/pmd.56
dat_100/pmd.58
dat_100/pmd.60
dat_100/pmd.62
dat_100/pmd.64
dat_100/pmd.66
dat_100/pmd.68
dat_100/pmd.70
dat_100/pmd.72
dat_100/pmd.74
dat_100/pmd.76
dat_100/pmd.78
dat_100/pmd.80
dat_100/pmd.82
dat_100/pmd.84
dat_100/pmd.86
dat_100/pmd.88
dat_100/pmd.90
dat_200/pmd.64
dat_200/pmd.66
dat_200/pmd.68
Density
0
1
2
3
4
5
6
−2
−1
0
1
2
3

A - 51.6 ns

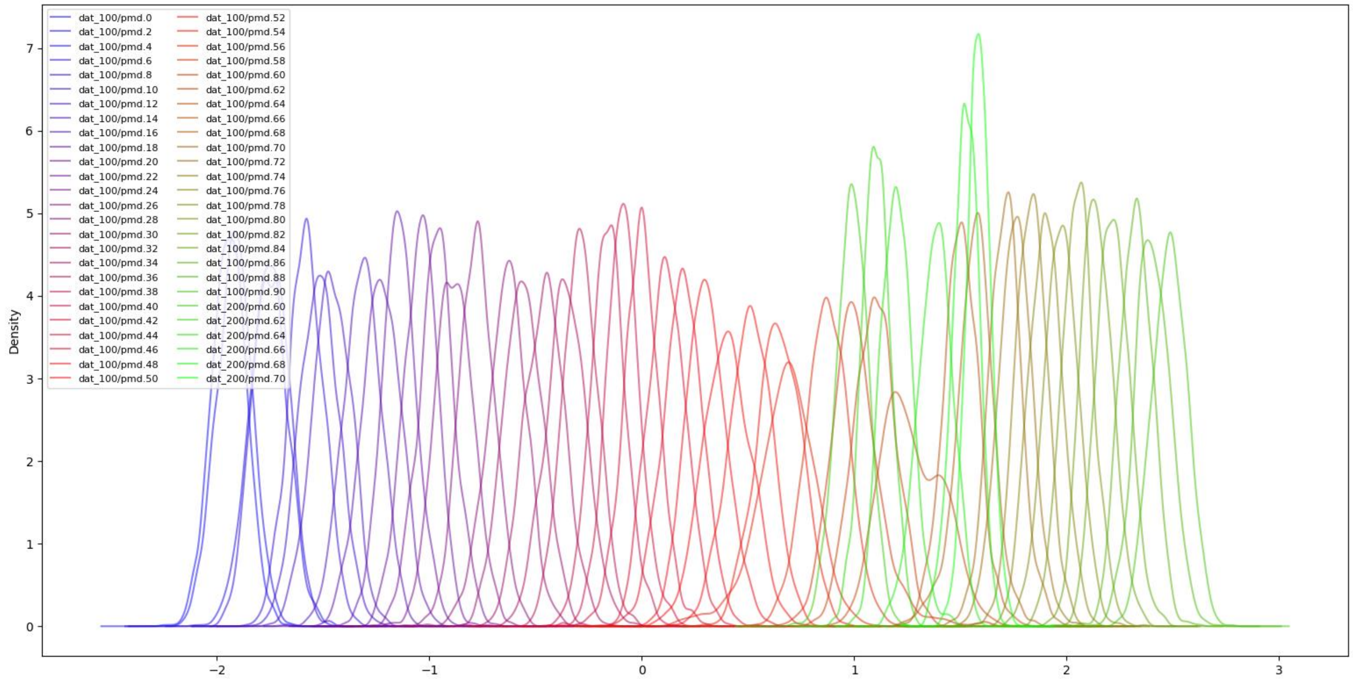

dat_100/pmd.0
dat_100/pmd.2
dat_100/pmd.4
dat_100/pmd.6
dat_100/pmd.8
dat_100/pmd.10
dat_100/pmd.12
dat_100/pmd.14
dat_100/pmd.16
dat_100/pmd.18
dat_100/pmd.20
dat_100/pmd.22
dat_100/pmd.24
dat_100/pmd.26
dat_100/pmd.28
dat_100/pmd.30
dat_100/pmd.32
dat_100/pmd.34
dat_100/pmd.36
dat_100/pmd.38
dat_100/pmd.40
dat_100/pmd.42
dat_100/pmd.44
dat_100/pmd.46
dat_100/pmd.48
dat_100/pmd.50
dat_100/pmd.52
dat_100/pmd.54
dat_100/pmd.56
dat_100/pmd.58
dat_100/pmd.60
dat_100/pmd.62
dat_100/pmd.64
dat_100/pmd.66
dat_100/pmd.68
dat_100/pmd.70
dat_100/pmd.72
dat_100/pmd.74
dat_100/pmd.76
dat_100/pmd.78
dat_100/pmd.80
dat_100/pmd.82
dat_100/pmd.84
dat_100/pmd.86
dat_100/pmd.88
dat_100/pmd.90
dat_200/pmd.60
dat_200/pmd.62
dat_200/pmd.64
dat_200/pmd.66
dat_200/pmd.68
dat_200/pmd.70
Density
0
1
2
3
4
5
6
7
−2
−1
0
1
2
3

B - 68.7 ns

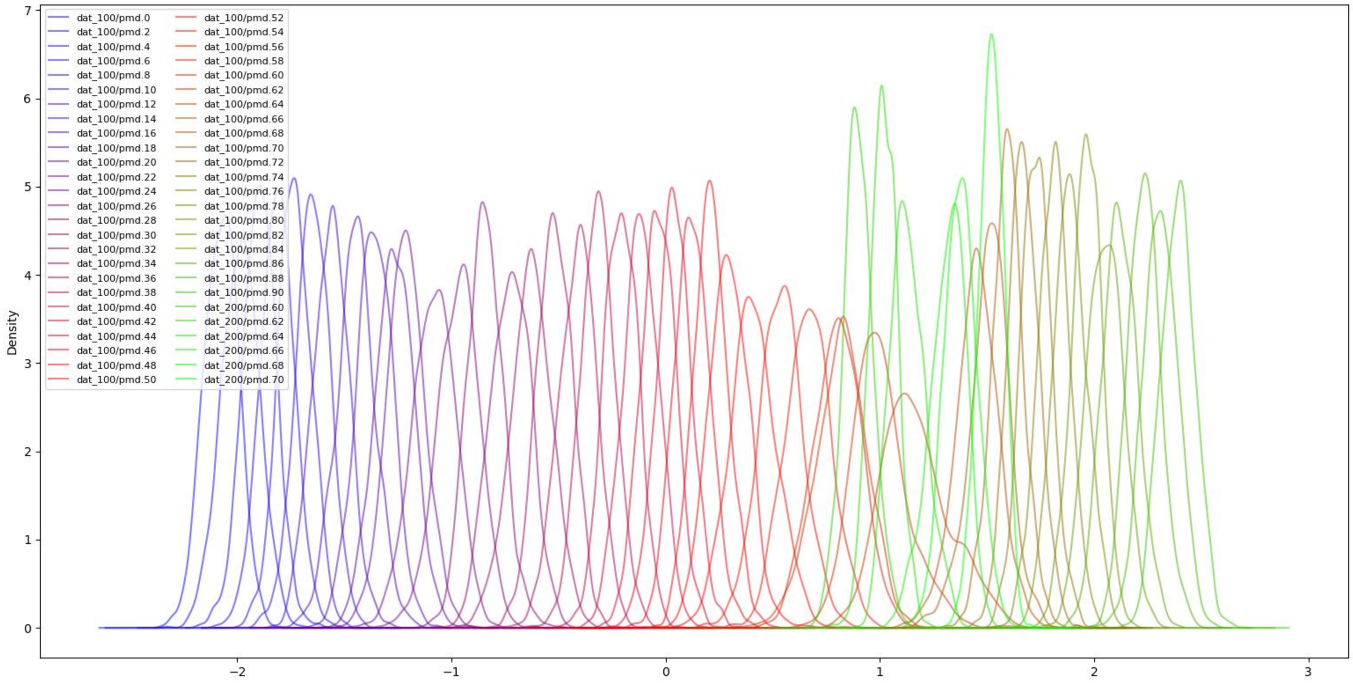

C - 85.7 ns

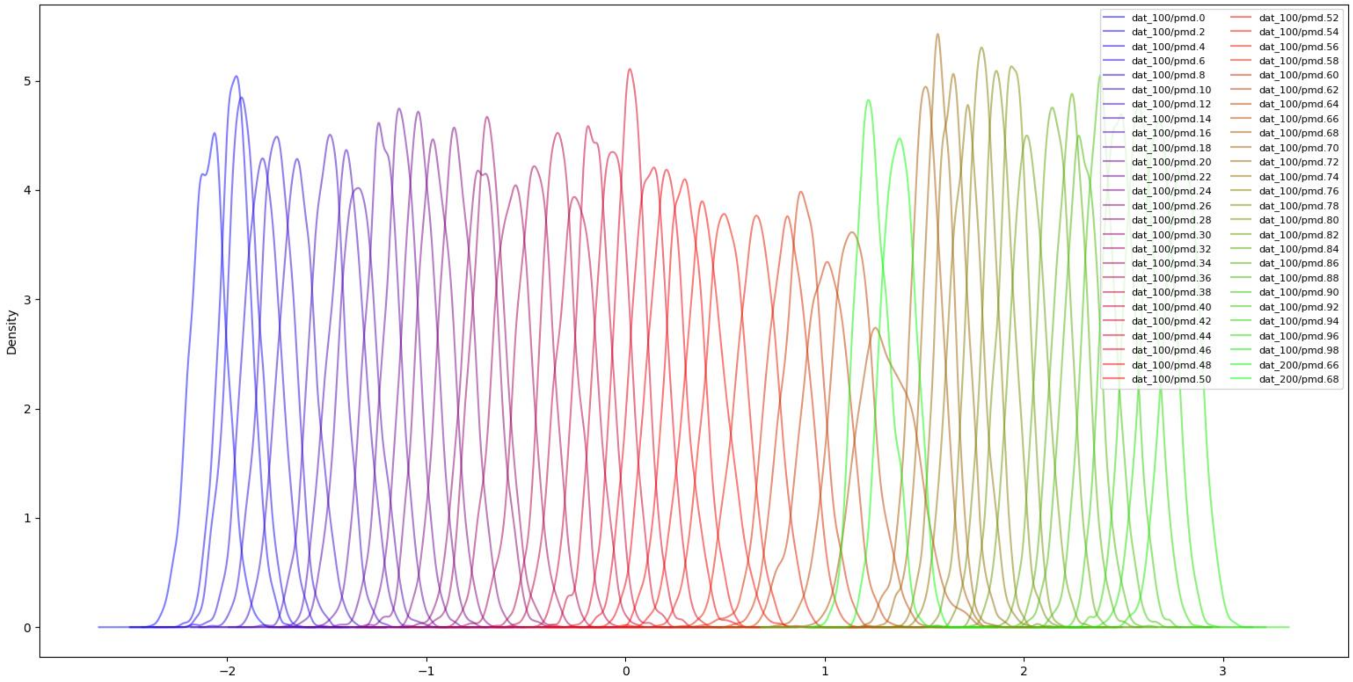

Figure S2. Density distributions for the collective variable $RC = d_{glyc} - d_{acid}$, for each umbrella simulation window during the 10 ps/window.

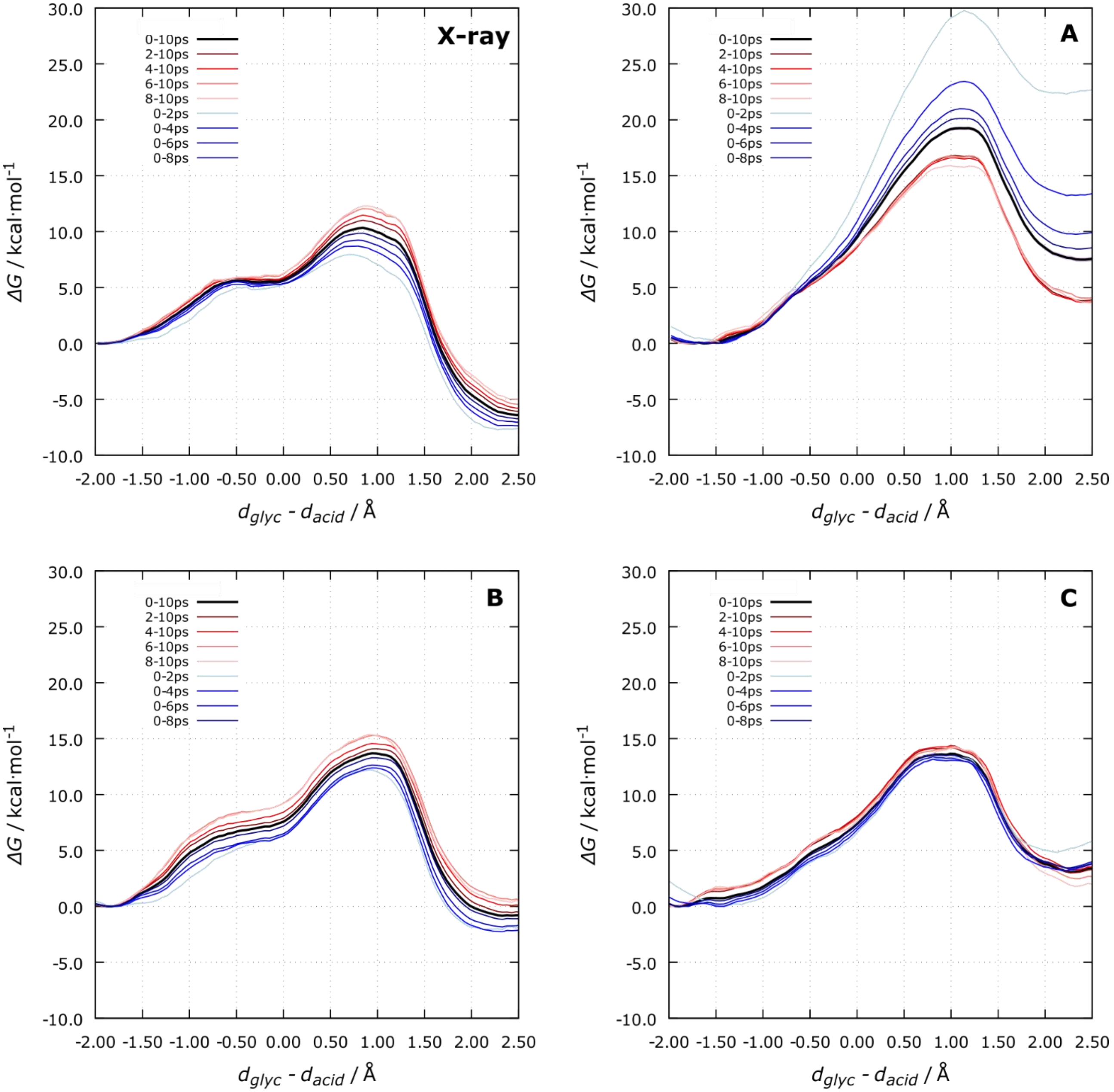

Figure S3. Gibbs energy profiles for the 4 selected starting conformations, for different timeseries across the 10 ps/window simulation. When considering the last 8 ps per US QM/MM MD window and a 1–2 kcal·mol^{-1} difference threshold between two consecutive Gibbs energy plots, the Gibbs barriers seem converged for every of the four starting conformations. Hence, the first 2 ps of each window were considered an equilibration period.

Enzyme:substrate dynamics

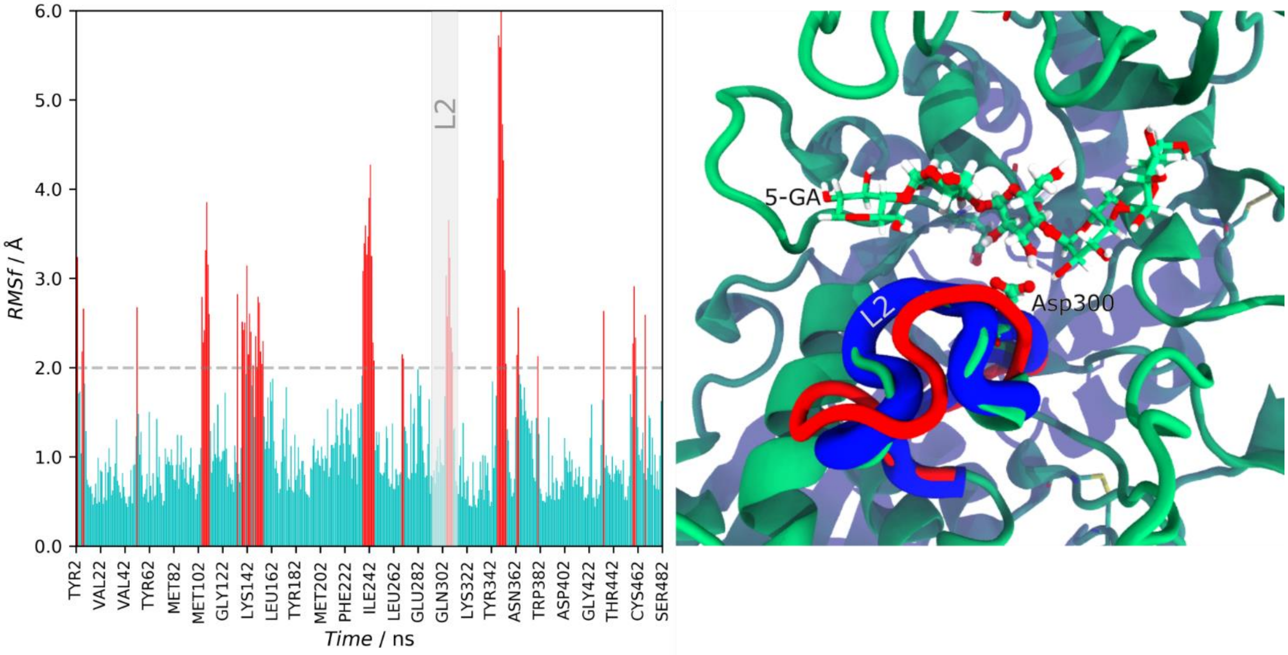

Figure S4. Left, root-mean-square fluctuation of enzyme residues along the 2 μs MD simulations; the L2 loop identified by Kosugi and Hayashi is highlighted. Right, representation of the two distinct conformations of the loop L2 along the MD simulations: the most populated cluster (~85% of the MD simulation), which is also the closest to the X-ray starting structure, is colored in blue, and the second most populated cluster (<15% of the MD simulation) is colored in red. The 5-GA substrate and the conserved Asp300, relevant for the catalysis, are also highlighted.

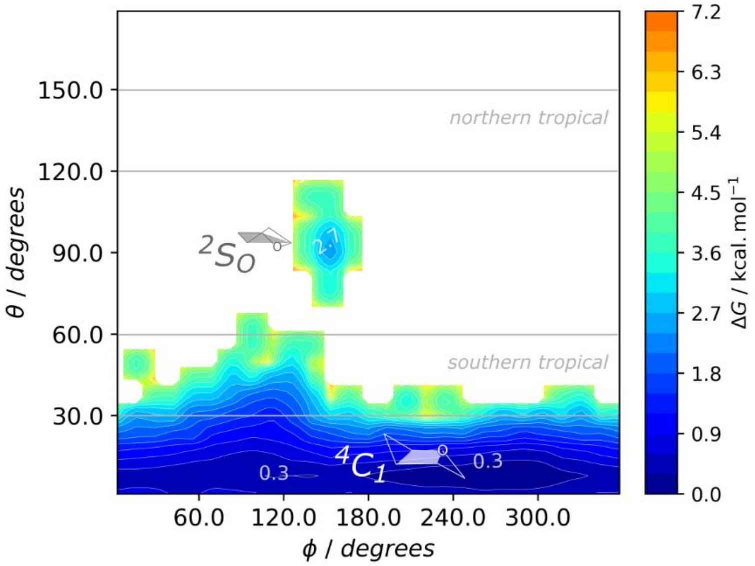

Figure S5. Projection of the Gibbs energy along the Cremer-Pople puckering angles φ and θ for the glucoside undergoing nucleophilic attack by Asp197, during the accumulated 2 μs cMD simulations. The puckering conformations found for the glucoside are highlighted: chair ($^4C_1$) and skew (^2S_O) conformations.

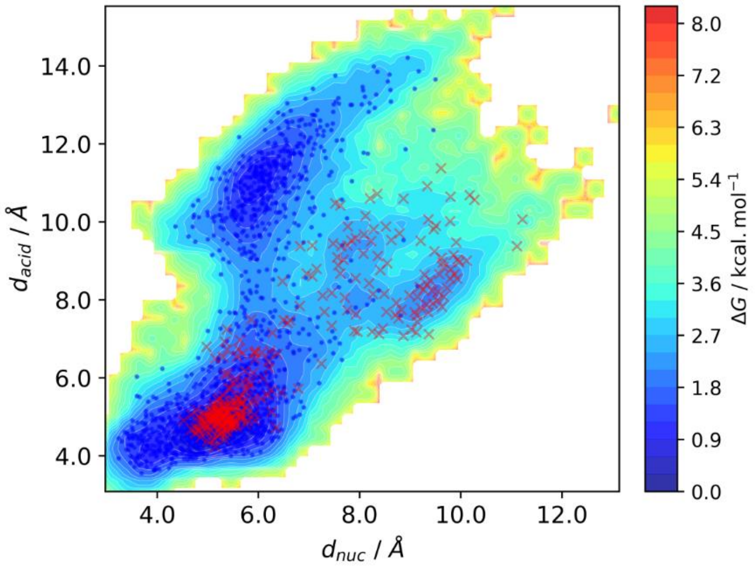

Figure S6. Gibbs energy plot of the 2 μs cMD projected on the space of the distances of the nucleophilic attack by Asp197 (d_{nuc}) and the acid-base reaction by Glu233 (d_{acid}), with blue dots and red crosses representing the conformations composing the clusters with 85% and 14% occupation after clustering analysis with the backbone atoms of the residues in loop L2.

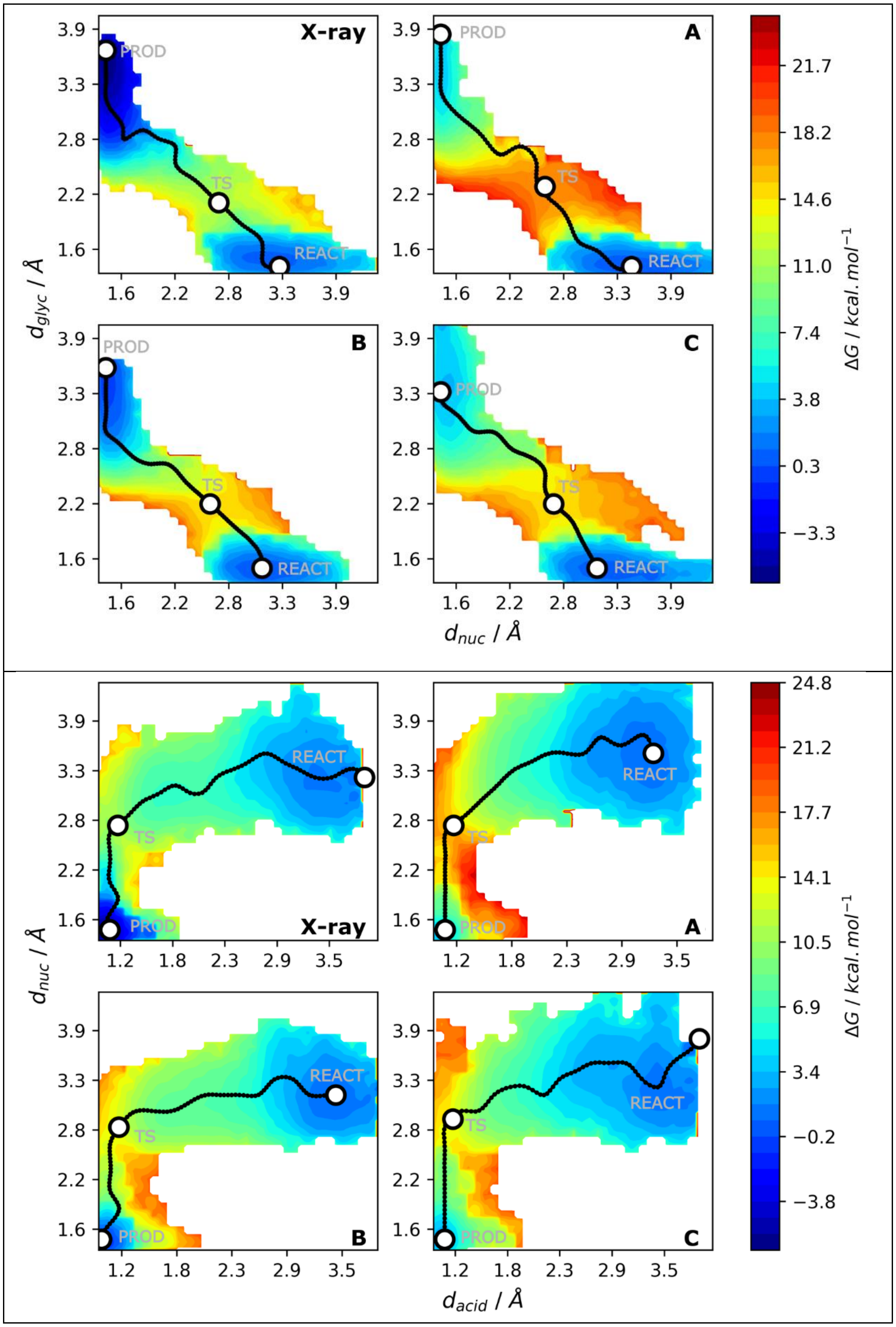

Figure S7. Representation of the distance of the nucleophilic Asp197 vs. the breaking glycosidic bond (top), and of the distance of the acid Glu233 vs. the nucleophilic Asp197 (bottom), for each independent frame where the reaction was carried out.

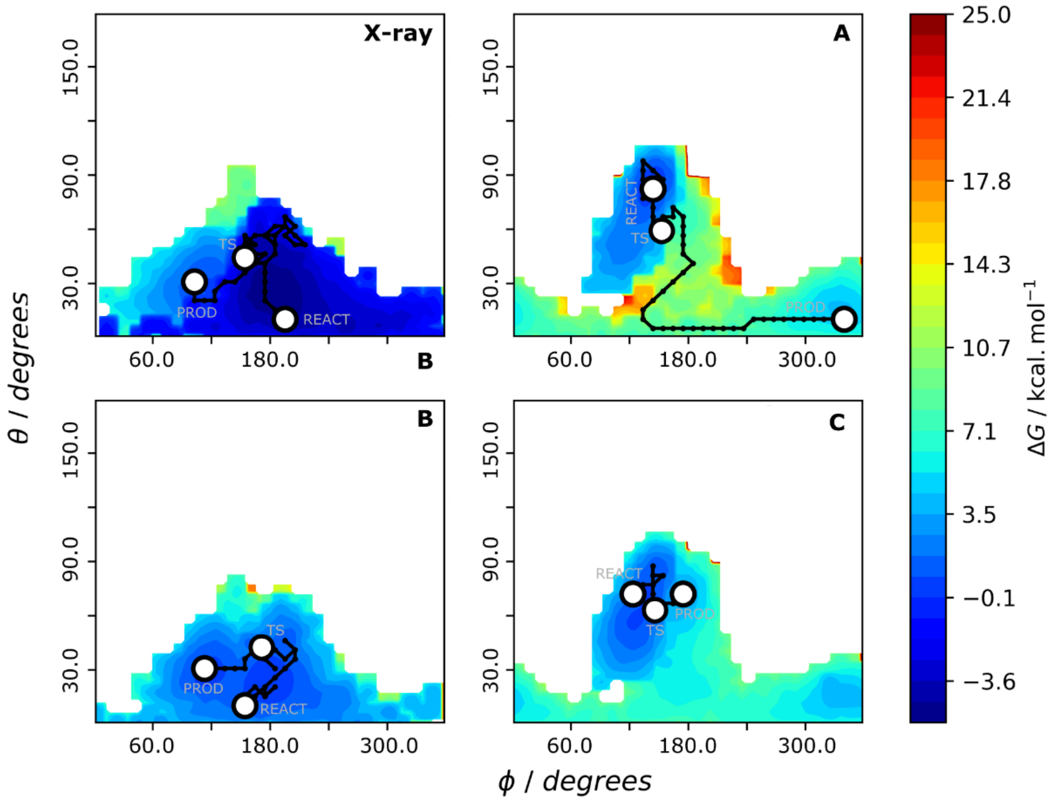

Figure S8. Representation of the Gibbs energy along the Cremer-Pople puckering angles φ and θ for the glucoside undergoing nucleophilic attack by Asp197, for each independent frame where the reaction was carried out.

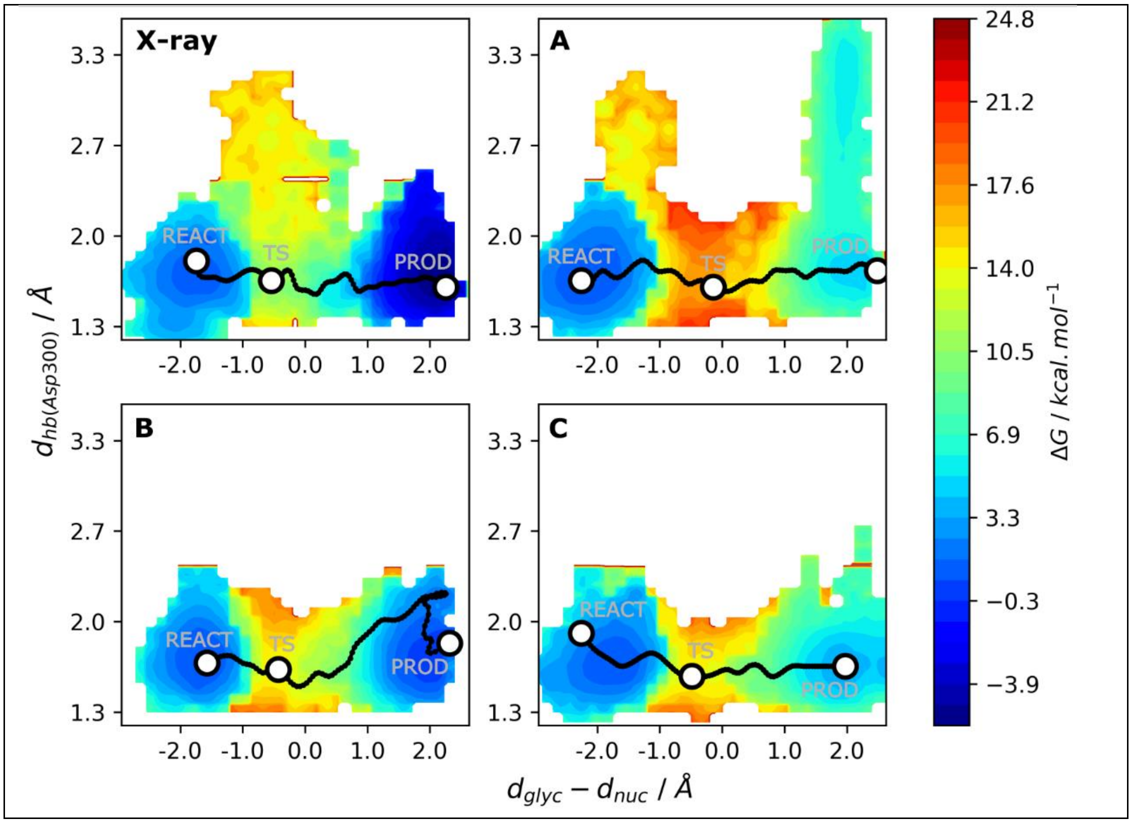

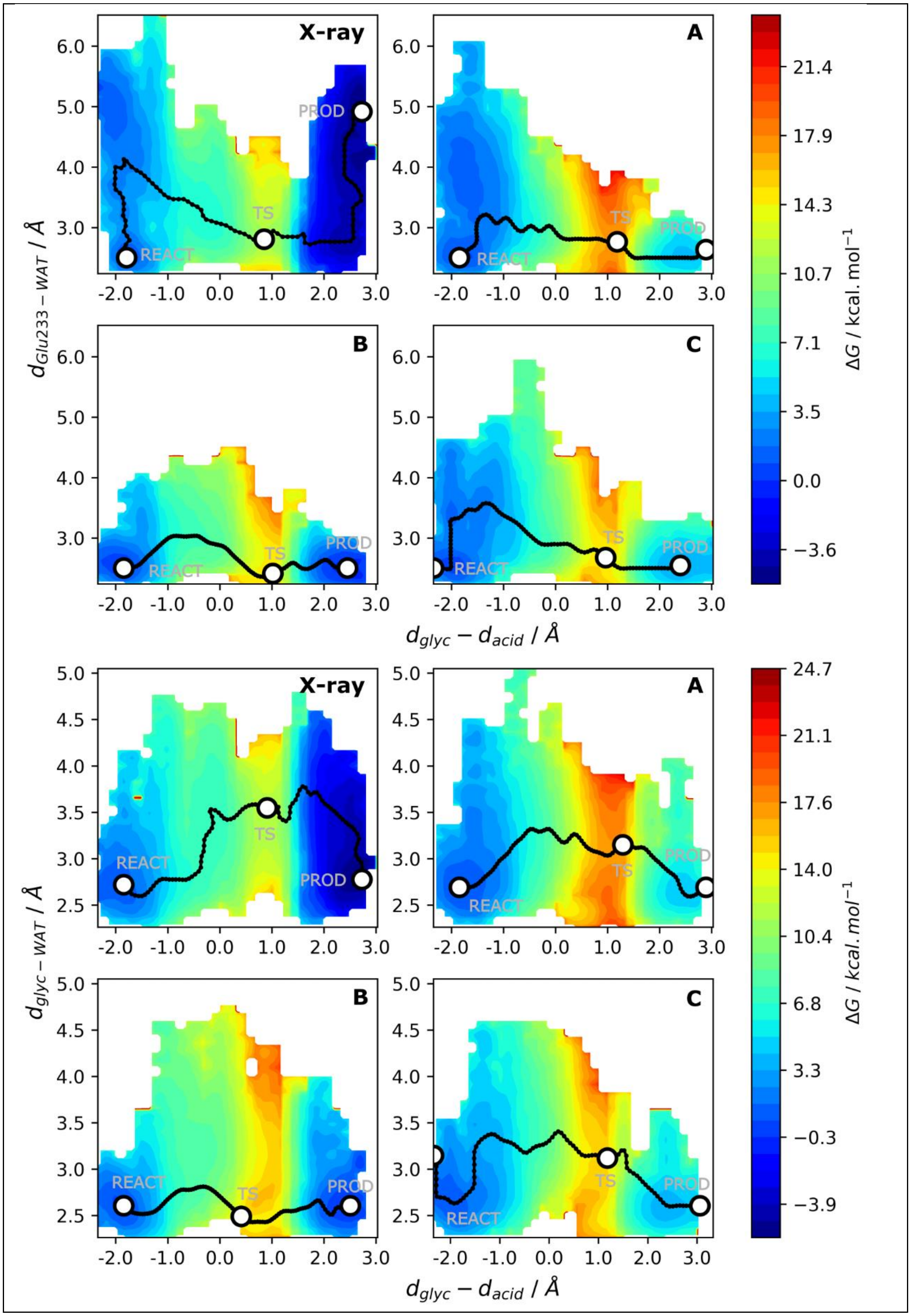

Figure S9. Representation of the distance of the Asp300-carboxylate vs. the guess reaction coordinate defined to study the glycosylation step, for each independent frame where the reaction was carried out (top); representation of the distance of the center of mass of the water molecule in the active site relatively to Glu233 and the glycosidic oxygen vs. the guess reaction coordinate defined to study the glycosylation step, for each independent frame where the reaction was carried out (bottom).

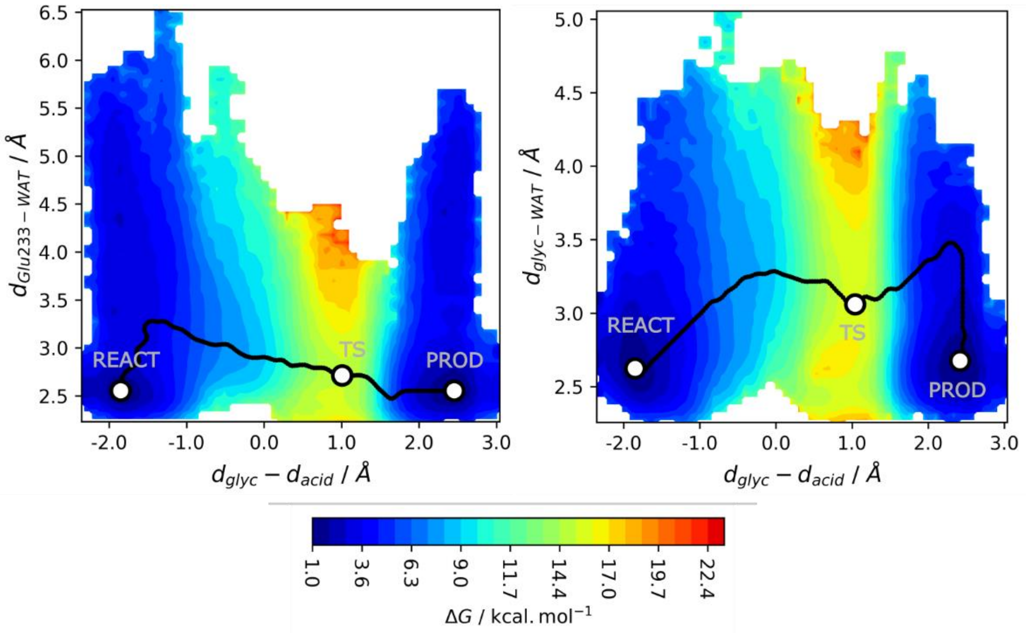

Figure S10. Distribution of distances corresponding to hydrogen bonds of the Glu233 and the glycosidic oxygen with a solvent water molecular along the guess reaction coordinate, during the 1.6 ns QM/MM MD simulations.

REFERENCES

1. Case, D. A.; Ben-Shalom, I. Y.; Brozell, S. R.; Cerutti, D. S.; Cheatham III, T. E.; Cruzeiro, V. W. D.; Darden, T. A.; Duke, R. E.; Ghoreishi, D.; Gilson, M. K.; Gohlke, H.; Goetz, A. W.; Greene, D.; Harris, R.; Homeyer, N.; Izadi, S.; Kovalenko, A.; Kurtzman, T.; Lee, T. S.; LeGrand, S.; Li, P.; Lin, C.; Liu, J.; Luchko, T.; Luo, R.; Mermelstein, D. J.; Merz, K. M.; Miao, Y.; Monard, G.; Nguyen, C.; Nguyen, H.; Omelyan, I.; Onufriev, A.; Pan, F.; Qi, R.; Roe, D. R.; Roitberg, A.; Sagui, C.; Schott-Verdugo, S.; Shen, J.; Simmerling, C. L.; Smith, J.; Salomon-Ferrer, R.; Swails, J.; Walker, R. C.; Wang, J.; Wei, H.; Wolf, R. M.; Wu, X.; Xiao, L.; York, D. M.; Kollman, P. A. *Amber 18*, University of California: San Francisco, 2018.
2. Hess, B., P-LINCS: A parallel linear constraint solver for molecular simulation. *J Chem Theor Comp* **2008,** *4* (1), 116-122.
3. Miyamoto, S.; Kollman, P. A., SETTLE: An Analytical Version of the Shake and Rattle Algorithm for Rigid Water Models. *J Comput Chem* **1992,** *13* (8), 952-962.
4. Ewald, P. P., The calculation of optical and electrostatic grid potential. *Ann Phys-Berlin* **1921,** *64* (3), 253-287.
5. Berendsen, H. J. C.; Postma, J. P. M.; Vangunsteren, W. F.; Dinola, A.; Haak, J. R., Molecular-Dynamics with Coupling to an External Bath. *J Chem Phys* **1984,** *81* (8), 3684-3690.
6. Bussi, G.; Donadio, D.; Parrinello, M., Canonical sampling through velocity rescaling. *J Chem Phys* **2007,** *126* (1), 014101.
7. Pinto, G. P.; Brás, N. F.; Perez, M. A. S.; Fernandes, P. A.; Russo, N.; Ramos, M. J.; Toscano, M., Establishing the Catalytic Mechanism of Human Pancreatic α-Amylase with QM/MM Methods. *J Chem Theor Comp* **2015,** *11* (6), 2508-2516.
8. Santos-Martins, D.; Calixto, A. R.; Fernandes, P. A.; Ramos, M. J., A Buried Water Molecule Influences Reactivity in a a-Amylase on a Subnanosecond Time Scale. *ACS Catal* **2018,** *8* (5), 4055-4063.
9. Pereira, A. T.; Ribeiro, A. J. M.; Fernandes, P. A.; Ramos, M. J., Benchmarking of density functionals for the kinetics and thermodynamics of the hydrolysis of glycosidic bonds catalyzed by glycosidases. *Int J Quantum Chem* **2017,** *117* (18), e25409.
10. Neese, F., Software update: the ORCA program system, version 4.0. *WIREs Comput Mol Sci* **2018,** *8* (1), e1327.
11. Biarnés, X.; Ardèvol, A.; Iglesias-Fernández, J.; Planas, A.; Rovira, C., Catalytic Itinerary in 1,3-1,4-β-Glucanase Unraveled by QM/MM Metadynamics. Charge Is Not Yet Fully Developed at the Oxocarbenium Ion-like Transition State. *J Am Chem Soc* **2011,** *133* (50), 20301-20309.
12. Alonso-Gil, S.; Coines, J.; Andre, I.; Rovira, C., Conformational Itinerary of Sucrose During Hydrolysis by Retaining Amylosucrase. *Front Chem* **2019,** *7*, 269.
13. Cuxart, I.; Coines, J.; Esquivias, O.; Faijes, M.; Planas, A.; Biarnés, X.; Rovira, C., Enzymatic Hydrolysis of Human Milk Oligosaccharides. The Molecular Mechanism of Bifidobacterium Bifidum Lacto-N-biosidase. *ACS Catal* **2022,** *12* (8), 4737-4743.
14. Teze, D.; Coines, J.; Fredslund, F.; Dubey, K. D.; Bidart, G. N.; Adams, P. D.; Dueber, J. E.; Svensson, B.; Rovira, C.; Welner, D. H., O-/N-/S-Specificity in Glycosyltransferase Catalysis: From Mechanistic Understanding to Engineering. *ACS Catal* **2021,** *11* (3), 1810-1815.
15. Neese, F., The ORCA program system. *WIREs Comput Mol Sci* **2012,** *2* (1), 73-78.
